# NarAB Is an ABC-Type Transporter That Confers Resistance to the Polyether Ionophores Narasin, Salinomycin, and Maduramicin, but Not Monensin

**DOI:** 10.3389/fmicb.2020.00104

**Published:** 2020-02-04

**Authors:** Ali-Oddin Naemi, Hymonti Dey, Nosheen Kiran, Sarah Torbergsen Sandvik, Jannice Schau Slettemeås, Live L. Nesse, Roger Simm

**Affiliations:** ^1^Institute of Oral Biology, Faculty of Dentistry, University of Oslo, Oslo, Norway; ^2^Norwegian Veterinary Institute, Oslo, Norway

**Keywords:** polyether ionophore, narasin, antimicrobial resistance, *Enterococcus faecium*, VRE

## Abstract

Polyether ionophores are antimicrobial compounds used as feed additives in poultry feed to control diseases caused by coccidia. In addition to the anticoccidial activity of these compounds, polyether ionophores also contain antibacterial properties. Resistance to the polyether ionophore narasin was recently shown to exist on mobile plasmids in *Enterococcus faecium* and the resistance mechanism was suggested to be associated with a two-gene operon encoding an ABC-type transporter. In this study we demonstrate that the genes encoding the putative narasin resistance mechanism confers reduced susceptibility to the polyether ionophores narasin, salinomycin and maduramicin, but not to monensin and suggest that this resistance mechanism should be referred to as NarAB. Importantly, NarAB does not affect the susceptibility of *E. faecium* to any of the tested antimicrobial compounds that are used in clinical medicine. However, we show that conjugation in the presence of certain polyether ionophores increases the number of vancomycin resistant *E. faecium* suggesting that narasin and certain other polyether ionophores can contribute to the persistence of VRE in poultry populations.

## Introduction

Polyether ionophores are used worldwide as feed additives in conventional rearing of poultry to control coccidiosis caused by the genus *Eimeria* (Kadykalo et al., 2018). In addition to the anticoccidial activity of polyether ionophores, they also possess antibacterial activity, mainly against Gram-positive bacteria. Until recently, it was assumed that there was no genetically encoded resistance mechanism to polyether ionophores, and that reduced susceptibility of bacteria to these compounds was due to a phenotypic adaptation (Simjee et al., 2012). However, it has been shown that resistance to the polyether ionophore narasin can be transferred between strains of enterococci by conjugation (Nilsson et al., 2012), proving the existence of a genetically encoded narasin resistance mechanism. Evidence has also emerged suggesting that narasin resistance might be associated with an operon encoding an ABC-type membrane transporter (Nilsson et al., 2016), but this has not yet been directly confirmed experimentally.

The polyether ionophores: monensin, narasin, maduramicin, salinomycin, and lasalocid are approved for use as feed additives in Norway (VKM, 2015). During the last decades, feed supplemented with monensin and narasin have been used for rearing of turkeys and broilers, respectively. In the Norwegian program for surveillance of antimicrobial resistance in the veterinary and food production sectors (NORM-VET), narasin has been included in the test panel for enterococci from different animals and food products. Surveillance data from 2002 to 2018 show that a high proportion of *Enterococcus faecium* isolates from poultry (25–91%) display reduced susceptibility to narasin (Norm/Norm-Vet, 2001–2014). Interestingly, all available vancomycin resistant *E. faecium* (VRE) from NORM-VET are also narasin resistant (Norm/Norm-Vet, 2001–2014). Narasin resistant bacteria as such are not considered to be a great problem, since narasin is currently not used in human medicine. However, it is not established if the narasin resistance mechanism offers cross-resistance to medically important antimicrobials. It has also been shown that narasin resistance and vancomycin resistance can be physically linked on transferrable plasmids (Sletvold et al., 2008; Nilsson et al., 2016), which indicates that use of narasin can co-select for vancomycin resistance. Vancomycin is used for the treatment of infections caused by Gram-positive bacteria (Alvarez et al., 2016), and is classified by WHO as a critically important antimicrobial for human medicine (WHO, 2017).

Vancomycin resistant *E. faecium* are a major cause of nosocomial infections worldwide and the incidence of serious infections caused by this opportunistic pathogen increases in many countries (Contreras et al., 2019). In the early 1990s, a community reservoir of VRE was discovered in Europe, which raised concern for the public health (van Belkun et al., 1996; Endtz et al., 1997). It is believed it emerged as a result of widespread use of the glycopeptide avoparcin in animal husbandry (Aarestrup, 1995; Klare et al., 1995a, b; Aarestrup et al., 2000). Consequently, avoparcin was banned as a feed additive in Norway and the EU in 1995 and 1997, respectively. For unclear reasons, a VRE reservoir still exists in animal populations in many European countries, more than 20 years after the avoparcin ban. However, the use of polyether ionophores as a feed additive overlapped and/or succeeded the use of avoparcin in poultry feed (Grave et al., 2004; Kaldhusdal et al., 2016). Considering the close association between narasin resistance and vancomycin resistance (Norm/Norm-Vet, 2001–2014; SVARM, 2011, 2013) it is possible that the use of narasin (or other polyether ionophores) in animal husbandry has contributed to maintaining the VRE reservoir in animals in Europe. Due to a decision by the Norwegian broiler industry, broilers have been reared without in-feed anticoccidials since 2016. This may have contributed to a dramatic reduction in narasin resistant isolates in broilers in 2018 (Norm/Norm-Vet, 2019). Turkeys on the other hand, still receive in-feed monensin and the proportion of narasin-resistant *E. faecium* isolates from turkeys remained on a high level in 2018 (Norm/Norm-Vet, 2019). This suggests that monensin promotes persistence of narasin-resistant *E. faecium* in turkeys and that the narasin resistance mechanism reduces the bacterial susceptibility to monensin. However, although resistance to monensin has been described in the literature (Newbold et al., 1993; Callaway and Russell, 1999; Houlihan and Russell, 2003), cross-resistance between monensin and narasin has not been detected (Butaye et al., 2000). This study characterizes the ABC-type transporter associated with narasin resistance, and demonstrates that it confers resistance to narasin, salinomycin and maduramicin, but not to monensin or selected antimicrobials currently used in human medicine. In addition, we show that polyether ionophores can influence the outcome of horizontal gene transfer of vancomycin resistance determinants, supporting the notion that they can contribute to spread of vancomycin resistance and persistence of VRE in poultry populations.

## Materials and Methods

### Description of Isolates

Eighteen *E. faecium* isolates were selected from the biobank of the Norwegian Veterinary Institute, representing different narasin minimum inhibitory concentration (MIC) values ranging from 1 to 32 mg/L. The 18 isolates were isolated in different years (sampled in 2004, 2006, or 2013) and from different geographical locations. Five of the isolates were also vancomycin-resistant and *vanA*-positive. The isolates had been susceptibility tested against a panel of antimicrobial compounds as part of the NORM-VET surveillance program (Table 1).

**TABLE 1:** Antimicrobial susceptibility of *E. faecium* isolates used in this study.

	**MIC (mg/L)**							
**Isolate**	**Van**	**Nar**	**Bac**	**Str**	**Gen**	**Tet**	**Lin**	**Amp**
WT15	>128	8	16	64	4	0.5	1	2
WT1137	1	8	256	64	16	1	4	2
WT1145	2	8	64	64	16	0.5	1	1
WT1147	1	8	128	32	8	0.5	4	2
WT1155	1	8	256	32	8	0.5	2	2
WT1165	1	4	256	64	8	1	4	2
WT1174	1	4	256	64	8	1	4	2
WT1179	2	8	256	64	8	64	4	0.5
WT1190	1	1	4	32	16	1	2	1
WT1251	2	4	32	256	256	1	n.d.	1
WT1301	1	8	16	256	256	1	n.d.	≤0.25
WT1343	1	8	8	256	256	1	n.d.	4
WT1366	1	8	2	256	256	1	n.d.	2
WT1402	>128	16	1	64	8	0.5	2	32
WT2608	1	1	32	32	8	0.5	2	2
WT4826	>128	8	4	16	8	0.5	0.5	≤0.25
WT5252	>128	32	1	64	8	0.5	2	0.5
WT5432	>128	8	1	32	4	0.5	1	4

*Van, vancomycin; Nar, narasin; Bac, bacitracin; Str, streptomycin; Gen, gentamicin; Tet, tetracycline; Lin, linezolid; Amp, ampicillin; n.d., not determined.*

### Detection of the Putative Narasin Resistance Mechanisms

DNA was extracted from the 18 isolates using NUCLISENS^®^ easyMAG^®^ (Biomerieux) according to the manufacturer’s description. The putative narasin resistance operon was detected using HotStarTaq polymerase (qiagen) in 25 μl PCR reactions containing 1X HotStarTaq Master mix, 0.2 μM of each primer and 2 μl DNA template using primers specific for the two genes (Supplementary Table 3). PCRs were run at 94°C for 15 min, 35 cycles of (94°C, 20 s; 63°C; 30 s; 72°C, 50 s), and 72°C, 10 min.

### Conjugation

In general, conjugation was performed by filter mating between a donor strain (the 18 wild type isolates selected from the biobank of the Norwegian Veterinary Institute), and the fusidic acid and rifampicin resistant plasmid free recipient strain *E. faecium* 64/3 (Bender et al., 2015). Cultures of the donors and the recipient were grown overnight in Brain heart infusion (BHI) broth to stationary phase at 37°C, and 150 rpm shaking. The donors were generally grown in the presence of narasin (1 mg/L), with the exception of the two strains with MIC values of narasin at 1 mg/L, these strains were grown in the presence of 0.5 mg/L narasin. The overnight cultures of the donors were washed once with BHI broth to remove narasin from the bacteria. The OD_600_ of the cultures was adjusted and donor and recipient were mixed at a 1 to 10 ratio and the bacteria were applied on filter membrane (0.22 μm GSWP, Merck) on BHI agar with or without relevant antimicrobials. Filter mating was performed at 37°C for 24 h. The bacteria were dislodged from the filter membranes and suspended in BHI broth, 10-fold serially diluted and plated on BHI agar supplemented with relevant antimicrobials.

Two different conjugation experiments were performed. In the first experiment, transfer of narasin resistance was tested using all 18 isolates from the Norwegian Veterinary Institute as donors. Filter mating was done on BHI agar without antimicrobials and the total number of bacteria with recipient background (i.e., recipient and transconjugants) and the number of transconjugants specifically, were quantified on BHI agar containing rifampicin (50 mg/L)/fusidic acid (25 mg/L) and narasin (1 mg/L)/rifampicin (50 mg/L)/fusidic acid (25 mg/L), respectively. Randomly selected colonies on the transconjugant-selective plates were stored and used for further experiments. Detection of narA, narB, and vanA was done by PCR using gene specific primers (Supplementary Table 3). The *narA* and *narB* genes were amplified using the PCR programs described under Section “Detection of the Putative Narasin Resistance Mechanisms,” whereas the *vanA* gene was amplified using the following program: 94°C for 15 min, 35 cycles of (94°C, 20 s; 58°C; 30 s; 72°C, 50 s), and 72°C, 10 min. In the second experiment WT1402, an isolate with co-transferrable narasin and vancomycin resistance, was used as donor to test if polyether ionophores can stimulate occurrence of vancomycin-resistant transconjugants. Filter mating was performed on BHI agar without antimicrobial supplementation or with narasin (0.25–8 mg/L), monensin (5 or 10 mg/L), maduramycin (16 mg/L), or salinomycin (4 mg/L). The total number of bacteria with recipient background and the number of transconjugants alone were analyzed on BHI agar supplemented with rifampicin (50 mg/L)/fusidic acid (25 mg/L) acid, and vancomycin (4 mg/L)/rifampicin (50 mg/L)/fusidic acid (25 mg/L), respectively. Randomly selected colonies found on the transconjugant selective plates were re-streaked on BHI agar supplemented with vancomycin (4 mg/L) to confirm growth of these isolates on 4 mg/L vancomycin. Vancomycin resistant transconjugants were also confirmed by MIC-testing using a broth microdilution assay.

### Sequencing and Bioinformatics Analysis

The annotated sequences of plasmids containing the putative narasin resistance operon were used to design sequencing primers (Supplementary Table 1). A subset of the 18 isolates representing different MIC values of narasin were chosen for sequencing. These isolates were: WT1145, WT1190, WT2608, WT1402, WT1251, WT1301, and WT1343. Sanger sequencing of the *narAB* operons was done at GATC biotech (Germany), and the sequences were assembled and compared by multiple sequence alignment using Clustal Ω (Madeira et al., 2019). The assembled sequences have been deposited into GenBank; accession numbers: MN590304-MN590310.

### Susceptibility Testing

A few bacterial colonies were picked from BHI agar plates, suspended in 1 mL of Mueller-Hinton (MH) medium, adjusted to OD 0.1 and then diluted 1000-fold in MH medium. The bacterial suspensions were distributed into the wells of 96-well round bottom plates and antimicrobials were added from twofold dilution series. The plates were incubated at 37°C for 48 h, results were registered every 24 h.

### Transcriptional Analysis

#### Growth Conditions

The expression of the putative narasin genes was analyzed using the transconjugants Tc1402 and Tc2608, and the recipient strain 64/3 as a control. For the transconjugants, one single colony was picked from BHI agar plate supplemented with narasin (1 mg/L), and for 64/3 one single colony was picked from a BHI agar plate without antimicrobial supplementation. The bacteria were suspended in 5 mL of BHI medium with (Tc1402, Tc2608) or without (64/3) narasin (1 mg/L). Following overnight incubation at 37°C, the OD_600_ of the cultures was adjusted to 0.01 in 5 mL BHI broth without antimicrobial supplementation. The cultures were grown to OD_600_ = 0.5. At this point narasin was added to the cultures to a final concentration of 1 mg/L and the cultures were continued for 20 min. The experiment was terminated by adding an equal volume of ice-cold methanol to the culture, followed by incubation for 5 min on ice. The bacteria were centrifuged for 5 min at 5000 *g* at 4°C, the supernatant was discarded and the pellet frozen at −20°C overnight.

#### RNA Extraction

Total RNA was isolated using the High Pure RNA Isolation Kit from Roche Life Science (cat# 11828665001) according to the manufacturer’s instructions for gram positive bacteria. The quality and purity of RNA were confirmed by analyzing the samples on a NanoDrop spectrophotometer (Thermo Fisher Scientific) and by running 1 μg of total RNA on a 1% agarose gel. Reverse transcriptase reactions were carried out on 1 μg of RNA using the First strand cDNA synthesis kit (Thermo Scientific) according to the manufacturer’s instructions. The cDNA samples were stored at −20°C until further analysis.

#### Quantitative PCR

The qPCR reactions were done with a total volume of 20 μL containing 10 μL of 2X PowerUp SYBR Green Mastermix (Applied Biosystems, Thermo Fisher Scientific), cDNA template (0.005 ng/μL) and a final concentration of 2 μM of forward and reverse primers. The qPCR was performed on AriaMX Real-Time PCR system with the following cycling conditions: 1 cycle for 2 min at 50°C (UDG activation), 1 cycle for 2 min at 95°C (Polymerase activation) and 40 cycles of 15 s denaturation at 95°C and 1 min annealing/extension at 60°C. Relative gene expression was determined using the ΔΔC_*q*_ method with *recA*, as a reference gene.

### Cloning of the Narasin Operon

The operon encoding the putative narasin resistance genes was amplified together with 906 nt of the upstream region from isolates WT1190, WT1402, and WT2608 using primers Nar-Reg-For and Nar-Reg-Rev (Supplementary Table 3). The PCR products and the plasmid pREG45 were digested with FastDigest *Bam*HI and FastDigest *Xho*I (Thermo Fisher Scientific) and the plasmid and insert were ligated together using rapid DNA ligation kit (Thermo Fisher Scientific). The ligation mixtures were introduced into competent DH5α as described by Chung et al. (1989) and bacteria were plated on LB-agar plates supplemented with spectinomycin (250 mg/L). Single colonies were screened for the presence of the *narAB* operon by colony PCR using primers: ABC_ATPase_For, ABC_ATPase_Rev, ABCpermease_For, ABCpermease_Rev (Supplementary Table 3) and the integrity of the constructs was confirmed by sanger sequencing at GATC Biotech (Germany), using primers: Nar-seq-rev1, Nar-seq-rev2, Nar-seq-rev3, Nar-seq-rev4, Nar-seq-rev5, Nar-seq-rev6, Nar-seq-for (Supplementary Table 3). The constructs were introduced into the plasmid free *E. faecium* SE34 (Teng et al., 1998) by electroporation using a previously published protocol (Grady and Hayes, 2003). Briefly, *E. faecium* SE34 were grown in M17 broth (Oxoid) overnight at 37°C. This culture was diluted 1:100 in 400 ml of M17 broth supplemented with 0.5M sucrose, containing 4% filter-sterilized glycine and grown for 16 h at 37°C. Bacteria were spun down at 4000 *g* for 10 min, and the cells were washed once with 20 ml of ice-cold electroporation buffer (0.5M sucrose, 10% glycerol). Aliquotes of electrocompetent cells were stored at −80°C. Plasmid DNA was electroporated into 100 μl of electrocompetent cells using an Gene Pulser electroporation system (Bio-Rad). Electroporation conditions were 2.5 kV, 0.2 cm cuvette gap, 25 μF and 200 ohm. Cells were diluted with 800 μl of Todd–Hewitt broth (Oxoid) supplemented with 0.5M sucrose and incubated at 37°C for 4 h before plating on BHI agar plates supplemented with spectinomycin (200 mg/L).

## Results

All 18 isolates selected for this study were shown by PCR to contain the genes encoding the putative narasin resistance mechanism (Supplementary Table 1 and Supplementary Figures 5, 6). The susceptibility data collected as part of the NORM-VET program (Table 1) was supplemented with MIC values of salinomycin, maduramicin, and monensin (Table 2).

**TABLE 2:** Susceptibility to polyether ionophores of *E. faecium* isolates used in this study.

	**MIC (mg/L)**				
**Isolate**	Nar*	**Sal**	**Mad**	**Mon**	**Las**
WT15	8	32	16	8	4
WT1137	8	8	32	2	4
WT1145	8	8	32	8	4
WT1147	8	8	32	2	4
WT1155	8	8	32	2	4
WT1165	4	16	4	16	4
WT1174	4	16	8	16	4
WT1179	8	32	16	16	4
WT1190	1	2	4	8	8
WT1251	8	8	32	32	4
WT1301	8	8	32	4	4
WT1343	8	8	32	4	4
WT1366	8	8	32	2	4
WT1402	16	32	32	8	2
WT2608	1	1	4	32	1
WT4826	4	8	32	8	2
WT5252	32	32	8	16	4
WT5432	8	32	8	4	4

Nar, narasin; Sal, salinomycin; Mad, maduramicin; Mon, monensin; Las, lasalocid. *The MIC of narasin was also shown in Table 1, but is included here to facilitate comparison.

In general, it appears that low and high MIC of narasin correlate with low and high MIC, respectively of salinomycin and maduramicin, but not monensin. There was no correlation between low and high MIC of narasin and low and high MIC of any of the other antimicrobial compounds (Table 1). These data indicate potential cross-resistance between narasin, salinomycin and maduramicin, but not between narasin and monensin, vancomycin, bacitracin, streptomycin, gentamicin, tetracycline, linezolid, or ampicillin.

### Sequencing of the Operon Encoding the ABC-Type Transporter and the Promoter Region

We hypothesized that the considerable variation in MIC of narasin among the isolates, even though they all were positive for the putative narasin resistance genes, were due to isolate-specific gene-variations. To test this, the relevant genes of a subset of the isolates representing different MIC values were sequenced. Multiple sequence alignment identified three single nucleotide polymorphisms giving rise to amino acid differences (Supplementary Figures 1–3), but none of them were representative of a certain MIC of narasin. Considering this, we speculated that differences in the promoter region could be responsible for the differences in narasin susceptibility. We attempted to amplify the upstream region of the same subset of isolates, however, we only managed to amplify and sequence the upstream region of three isolates. These three isolates WT1190, WT1402, and WT2608, displayed narasin MIC values of 1, 16, and 1 mg/L, respectively. Isolates WT1190 and WT2608 had identical upstream regions, but the region of these isolates differed compared to isolate WT1402 by 19 nucleotide substitutions and one nucleotide deletion (Supplementary Figure 4), indicating that the differences in narasin MIC might be due to differences in regulation of gene expression.

### Conjugation and MIC Testing of Transconjugants

**FIGURE 1:**
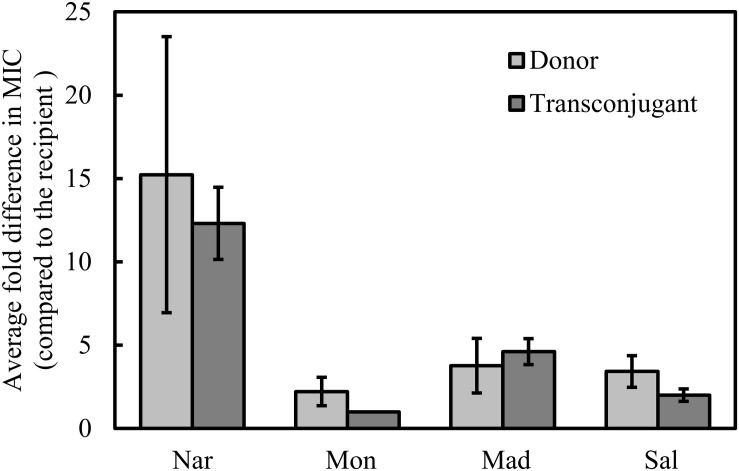
Relative MIC of polyether ionophores. MIC values were determined for the wild type isolates (donor) and the transconjugants and normalized against the MIC values displayed by the recipient strain. Shown are average values of 14 isolates/transconjugants and 95% confidence intervals. Nar, narasin; Mon, monensin; Mad, maduramicin; Sal, salinomycin.

It has previously been shown that narasin resistance can be transferred between strains of *E. faecium* by conjugation. We performed conjugation experiments using the 18 isolates as donors and selected for narasin resistant isolates. Conjugation was successful for 14 of the 18 isolates. Broth microdilution tests confirmed that susceptibility to narasin was decreased 8–16-fold in the transconjugants compared to the recipient strain (Figure 1 and Table 3). The *narA* and *narB* genes were detected in all transconjugants, but not in the recipient strain 64/3 (Supplementary Figures 7, 8 and Supplementary Table 2).

**TABLE 3:** Antimicrobial resistance of *E. faecium* 64/3 and transconjugants.

	**Antimicrobial compound (mg/L)**										
	
**Strain**	**Van**	**Nar**	**Mon**	**Mad**	**Sal**	**Bac**	**Str**	**Gen**	**Tet**	**Lin**	**Amp**
64/3	0.5	0.5	5	4	4	16	256	2	4	4	4
TC15	0.5	4	5	16	8	>256	256	2	4	4	4
TC1137	0.5	8	5	16	8	>256	256	4	4	4	4
TC1145	0.5	8	5	16	8	16	256	2	4	4	4
TC1147	0.5	8	5	16	16	16	256	4	4	4	4
TC1165	0.5	8	5	32	8	>256	512	2	4	4	4
TC1174	0.5	8	5	16	8	>256	256	2	4	4	4
TC1179	0.5	4	5	16	4	>256	256	2	4	4	4
TC1190	0.5	4	5	16	8	>256	256	16	4	4	4
TC1402*	256	8	5	32	8	16	256	4	4	4	4
TC2608	0.5	4	5	16	8	64	256	2	4	4	4
TC4826	0.5	4	5	16	8	16	256	2	4	4	4
TC5252*	256	8	5	32	8	16	512	2	4	4	4
TC5432	0.5	4	5	16	4	512	>256	2	4	4	4

Van, vancomycin; Nar, narasin; Mon, monensin; Mad, maduramicin; Sal, salinomycin; Bac, bacitracin; Str, streptomycin; Gen, gentamicin; Tet, tetracycline; Lin, linezolid; Amp, ampicillin. *vanA positive isolate. Strain 64/3 is the recipient. TC, transconjugant; the number corresponds to the wild type donor number.

To determine if the narasin resistance mechanism also can confer resistance to other antimicrobial compounds, the susceptibility of the transconjugants was tested against a panel of seven relevant antimicrobials (Table 3). Reduced susceptibility to clinically used antimicrobials was occasionally transferred from the donor strain. Importantly, vancomycin resistance was transferred from two of the five vancomycin resistant isolates. The vanA gene was detected in both transconjugants, but not in the recipient strain 64/3 (Supplementary Figure 9 and Table 2). We also tested the resistance profile of the transconjugants against polyether ionophores (Table 3) and compared it to the corresponding resistance profile of the donor and recipient strains (Figure 1). The resistance profiles of the transconjugants indicate that they are of the recipient background, this was however not confirmed by molecular methods.

Resistance to narasin in the transconjugants correlated with reduced susceptibility to maduramicin, and salinomycin, but not to monensin (Figure 1). It is noteworthy that the MIC of the polyether ionophores varied more in the donors than it did in the transconjugants (Figure 1).

### The Operon Encoding the ABC-Type Transporter Is Responsible for Narasin Resistance

To confirm that resistance to narasin was due to the ABC-type membrane transporter, we cloned the operon encoding it together with the native promoter from WT1402 and WT2608 into the vector pREG45, creating constructs pNAR1402 and pNAR2608. Both these constructs conferred reduced susceptibility to narasin compared to the vector control (Table 4). We also tested resistance against vancomycin, salinomycin, maduramicin, and monensin.

**TABLE 4:** Susceptibility to polyether ionophores and vancomycin.

	**Antimicrobial**				
	**compound (mg/L)**				
**Strain**	**Van**	**Nar**	**Mon**	**Sal**	**Mad**
SE34 (pREG45)	0.5	2	20	8	32
SE34 (pNAR1402)*	0.5	16	20	64	256
SE34 (pNAR2608)*	0.5	16	20	64	256

Van, vancomycin; Nar, narasin; Mon, monensin; Sal, salinomycin; Mad, maduramicin. *The upstream region of these constructs differ by 20 single nucleotide polymorphisms.

This experiment confirmed the previous data that the ABC-type transporter reduces the susceptibility of *E. faecium* against salinomycin and maduramicin, but not to monensin. Interestingly, the increases in MIC of narasin, salinomycin, maduramicin were identical for pNAR1402 and pNAR2608.

### Narasin Induces the Expression of the Narasin Resistance Operon

**FIGURE 2:**
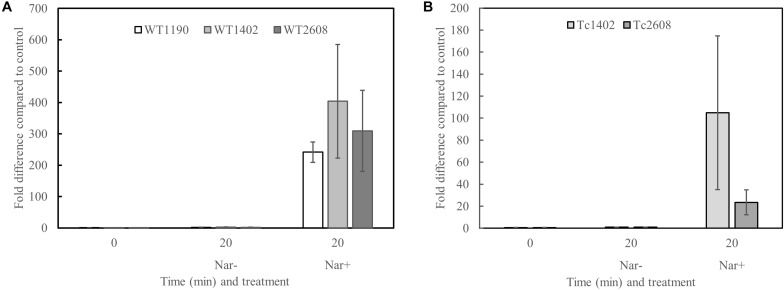
Narasin induces transcription of the putative narasin resistance genes. Bacteria were grown in BHI broth to mid-exponential phase. Narasin (1 mg/L) was added and incubation continued for 20 min. The narasin induced transcription was normalized to an uninduced parallel control. RT-qPCR was performed on mRNA from three independent experiments. Transcription of *narB* compared to *recA* in wild type isolates **(A)** and transconjugants **(B)**. Shown are averages and 95% confidence intervals. The higher expression in WT1402 compared to WT1190 and WT2608, and in TC1402 compared to TC2608 is not statistically significant (Student’s *t*-test; *p* > 0.05).

It is possible that narasin induces expression of the narasin resistance mechanism. To test this, we performed RT-qPCR analysis of *E. faecium* before and after exposure to narasin (Figure 2). Transcription of the narasin resistance genes increased slightly during exponential growth in the absence of narasin. However, addition of narasin induced expression in WT1190, WT1402, and WT2608 (Figure 2A) as well as in Tc2608 and Tc1402 (Figure 2B).

### Certain Polyether Ionophores Stimulate the Number of Vancomycin Resistant Transconjugants

**FIGURE 3:**
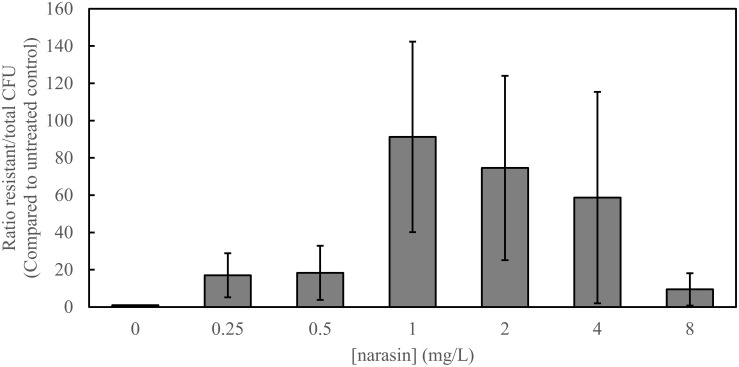
Narasin increases the number of vancomycin-resistant transconjugants. Filter mating was performed overnight, in the absence and presence of different concentrations of narasin. Ten-fold serial dilutions of bacteria were plated on BHI-agar supplemented or not with vancomycin (4 mg/L). The ratio of vancomycin-resistant and susceptible colony forming units (CFU) was calculated. Shown are average values and 95% confidence intervals of at least five independent experiments.

Since vancomycin resistance and narasin resistance can transfer simultaneously between strains of *E. faecium* (Nilsson et al., 2012; Table 3), we tested if polyether ionophores influence the occurrence of vancomycin-resistant transconjugants. For this purpose, we performed conjugation experiments between a vancomycin-resistant and narasin-resistant donor (WT1402) and a susceptible recipient (64/3) and analyzed the number of vancomycin-resistant transconjugants. The conjugation experiment was performed in the presence of different concentrations of narasin ranging from 0.25 to 8 mg/L and compared with control conditions without narasin. For comparison, the MIC value of narasin for the recipient was of 0.5 mg/L and the donor and transconjugant had MIC values of 16 and 8 mg/L, respectively (Tables 1, 2). The occurrence of vancomycin-resistant transconjugants varied in a narasin concentration dependent fashion (Figure 3).

**FIGURE 4:**
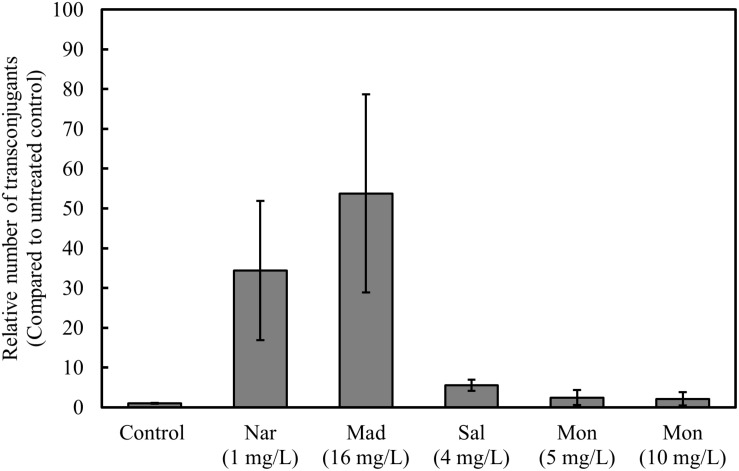
Monensin does not increase the number of vancomycin-resistant transconjugants. Filter mating was performed overnight, in the presence and absence of narasin, maduramicin, salinomycin, and monensin. Ten-fold serial dilutions of bacteria were plated on BHI-agar supplemented or not with vancomycin (4 mg/L). The ratio of vancomycin-resistant and susceptible CFU was calculated. Average values and 95% confidence intervals from at least five independent experiments are shown.

These results prompted a conjugation experiment to test the effect of other polyether ionophores on occurrence of vancomycin-resistant transconjugants. The occurrence of vancomycin resistant transconjugants significantly increased in the presence of maduramicin, and salinomycin, but not monensin (Figure 4).

## Discussion

Narasin resistance has been associated with a region on large transferrable plasmids encoding an uncharacterized ABC-type membrane transporter (Nilsson et al., 2016). In this study, we performed the first detailed characterization of this putative efflux pump and show that expression of the operon encoding it is induced by narasin, and that these genes are sufficient for achieving reduced susceptibility to narasin. We therefore suggest that the ATPase and permease subunits should be referred to as NarA and NarB, respectively and that the narasin resistance mechanism should be designated NarAB.

Previous publications report attempts to characterize polyether ionophore resistance mechanisms. The *narAB* operon occurs on pVEF3 (Nilsson et al., 2016), a sequenced and partially characterized plasmid from an *E. faecium* strain isolated from poultry in Norway (Sletvold et al., 2008). Sletvold et al. (2008) cloned a putative ABC-type transporter encoded by an operon located between nucleotides 4658 and 6675 on pVEF3. The authors did not detect activity conferring resistance to any of the 27 tested antimicrobial compounds by this construct in *E. faecium*. However, the *narAB* operon of pVEF3 is located between 37290 and 39808 (Sletvold et al., 2008; Nilsson et al., 2016), and this operon was never characterized. A resistance mechanism against a polyether ionophore was identified in 1994 when a genomic library of *Streptomyces longisporoflavus* was screened in *Streptomyces lividans* for tetronasin resistance determinants (Linton et al., 1994). This screen identified an operon containing the genes *tnrB2* and *tnrB3*, encoding an ABC-type membrane transporter that might be involved in self-resistance in the tetronasin producing bacterium. The tetronasin resistance mechanism has not been further characterized. Monensin resistance can develop in bacteria upon exposure to the antimicrobial compound (Newbold et al., 1993; Callaway and Russell, 1999; Houlihan and Russell, 2003). Monensin resistance has been attributed to thickening of the cell envelope and appears to be reversible when bacteria are growing in the absence of monensin. So far, monensin resistance has not been linked to a genetic determinant.

The *E. faecium* isolates included in this study are all positive for the *narAB* operon, but display different susceptibility to narasin. Analyses of the DNA sequences of *narA* and *narB* and the corresponding amino acid sequences did not reveal essential differences that could explain the observed differences in narasin susceptibility. Analyses of the upstream regions of *narAB* from three isolates revealed 20 nucleotide differences that could affect transcription of *narAB*. In line with this, our transcriptional analysis displayed a trend of higher transcription of *narAB* in the WT1402 compared to WT1190 and WT2608 and in Tc1402 compared to Tc2608, however, the difference in expression was not statistically significant (Student’s *t*-test; *p* > 0.05). We observed a twofold difference in MIC of narasin between Tc1402 and Tc2608. Based on these results, we cannot rule out that differences in the upstream region of *narAB* affects expression of the resistance mechanism. The difference in MIC of narasin between WT1402 and WT2608 is bigger than the difference in narasin MIC between Tc1402 and Tc2608 suggesting that not only the promoter region, but also the genetic background of the *E. faecium* strains affect the narasin susceptibility. Interestingly, when the *narAB* operons of WT1402 and WT2608 were cloned together with the upstream regions into pREG45, the constructs conferred identical narasin susceptibility of SE34. This may indicate that unknown factors on the respective wild type plasmids encoding *narAB* can influence the susceptibility to narasin.

It has previously been suggested that narasin resistance could be linked with resistance to medically important antimicrobials (Norm/Norm-Vet, 2001–2014; Nilsson et al., 2012, 2016), and a physical linkage between narasin resistance and vancomycin resistance has been demonstrated (Sletvold et al., 2008; Nilsson et al., 2016). In addition, a statistically significant correlation between narasin resistance and bacitracin resistance has been reported (VKM, 2015). However, we clearly show that NarAB does not directly confer resistance to any of the tested clinically used antimicrobials. This indicates that observed epidemiological association between narasin resistance and resistance to clinically used antimicrobials is likely due to co-occurrence of multiple resistance mechanisms in the same isolate. On the other hand, NarAB confer increased tolerance to salinomycin and maduramicin, demonstrating that the resistance mechanism is not specific for narasin. Cross-resistance between narasin and salinomycin has previously been shown in *E. faecium* isolates from farm animals (Butaye et al., 2000, 2001). Remarkably, NarAB does not affect the susceptibility of *E. faecium* (SE34) to monensin. This is interesting since surveillance data from NORM-VET demonstrated high prevalence of narasin resistant *E. faecium* in the Norwegian turkey population that receive monensin, but not narasin as a feed additive (Norm/Norm-Vet, 2019). In addition, we have previously shown that broiler feed, free of antimicrobial compounds significantly reduces the narasin-resistant population of *E. faecium* compared to narasin containing feed (Simm et al., 2019). This raises the question why narasin-resistant variants constitute a large proportion of the *E. faecium* population in turkeys, since there does not seem to be an obvious selection pressure.

We showed in a previous study that all VRE available in the biobank of the Norwegian Veterinary Institute are also resistant to narasin (Simm et al., 2019). It has also been shown that the resistance mechanisms to narasin and vancomycin can exist on the same plasmids and can be co-transferred between *E. faecium* strains (Sletvold et al., 2008; Nilsson et al., 2012, 2016). We show that in two of five cases, narasin resistance and vancomycin resistance co-existed in the transconjugants following conjugation between a vancomycin and narasin resistant donor and a vancomycin and narasin susceptible recipient. The resistance profiles of the transconjugants indicate that vancomycin and narasin resistance were co-transferred from VRE isolates to a plasmid-free *E. faecium* strain (Table 3). However, the recipient background of the transconjugants was not confirmed by molecular methods, which is a weakness of the experiment. Nonetheless, the experiment supports the previous findings that vancomycin and narasin resistance determinants occasionally co-exist on transferrable plasmids (Nilsson et al., 2012), and strengthen the hypothesis that narasin can promote persistence of vancomycin resistance in the *E. faecium* population (Simm et al., 2019). To further corroborate this notion narasin stimulated the occurrence of vancomycin resistant transconjugants in a concentration dependent manner (Figure 3). Interestingly, this was also the case for salinomycin and maduramicin, but not monensin, indicating that the resistance mechanism NarAB supports horizontal gene transfer under relevant selection pressure. Thereby, the use of in-feed narasin, salinomycin, and maduramicin may not only promote persistence of VRE in the *E. faecium* population of animals, but also promote dissemination of vancomycin resistance in the gut-microbiota of animals. Considering this, the justification for the widespread use of polyether ionophores as additives in animal feed should be re-evaluated.

## Data Availability Statement

The datasets generated for this study can be found in the NCBI, accession numbers: MN590304–MN590310.

## Author Contributions

RS conceived and designed the work. A-ON, HD, NK, SS, JS, and RS contributed to the acquisition of data. A-ON, HD, NK, SS, JS, LN, and RS contributed to the analysis and interpretation of data. RS wrote the first draft of the manuscript. A-ON, HD, JS, LN, and RS wrote sections of the manuscript. All authors contributed to manuscript revision, read, and approved the submitted version.

## Conflict of Interest

The authors declare that the research was conducted in the absence of any commercial or financial relationships that could be construed as a potential conflict of interest.
