# Supplementary material for: NarAB Is an ABC-Type Transporter That Confers Resistance to the Polyether Ionophores Narasin, Salinomycin, and Maduramicin, but Not Monensin
Source: Front Microbiol. 2020 Feb 4;11:104. doi: 10.3389/fmicb.2020.00104 (PMC7010723; doi:10.3389/fmicb.2020.00104)
Supplement: Supplementary file 1 [file Data_Sheet_1.docx]

Supplementary Material

# Supplementary Figures and Tables:

Supplementary Figure 1: Alignment of the nucleotide sequences of *narAB* operon

Supplementary Figure 2: Alignment of amino acid sequences of NarA

Supplementary Figure 3: Alignment of the amino acid sequences of NarB

Supplementary Figure 4: Supplementary Figure 4. The promoter region of *narAB*

Supplementary Table 1: Occurence of *narA* and *narB* in the wild type isolates included in the study

Supplementary Figure 5: Gel pictures showing PCR products of *narA* in wild type isolates

Supplementary Figure 6: Gel pictures showing PCR products of *narB* in wild type isolates

Supplementary Table 2: Detection of *narA, narB* and *vanA* in transconjugates, 64/3 and SE34

Supplementary Figure 7: PCR products of *narA* in transconjugants, 64/3 and SE34

Supplementary Figure 8: PCR products of *narB* in transconjugants, 64/3 and SE34

Supplementary Figure 9: PCR products of *vanA* in transconjugants, 64/3 and SE34

Supplementary Table 3: Primers used in this study

**Supplementary Figure 1. Alignment of the nucleotide sequence of *narAB***

WT1343 ATGACAGAAATTGTAAAAGTACAAGGCTTGCAAAAAAAATTTGGTAAATTCCAGGCGTTG 60

WT1301 ATGACAGAAATTGTAAAAGTACCAGGCTTGCAAAAAAAATTTGGTAAATTCCAGGCGTTG 60

WT1145 ATGACAGAAATTGTAAAAGTACAAGGCTTGCAAAAAAAATTTGGTAAATTCCAGGCGTTG 60

WT2608 ATGACAGAAATTGTAAAAGTACAAGGCTTGCAAAAAAAATTTGGTAAATTCCAGGCGTTG 60

WT1402 ATGACAGAAATTGTAAAAGTACAAGGCTTGCAAAAAAAATTTGGTAAATTCCAGGCGTTG 60

WT1190 ATGACAGAAATTGTAAAAGTACAAGGCTTGCAAAAAAAATTTGGTAAATTCCAGGCGTTG 60

WT1251 ATGACAGAAATTGTAAAAGTACAAGGCTTGCAAAAAAAATTTGGTAAATTCCAGGCGTTG 60

********************** *************************************

WT1343 AAAGATGTCTCATTCACAGTAAACGCCGGTGAAGTTGTTGGTTTTATCGGACCAAATGGA 120

WT1301 AAAGATGTCTCATTCACAGTAAACGCCGGTGAAGTTGTTGGTTTTATCGGACCAAATGGA 120

WT1145 AAAGATGTCTCATTCACAGTAAACGCCGGTGAAGTTGTTGGTTTTATCGGACCAAATGGA 120

WT2608 AAAGATGTCTCATTCACAGTAAACGCCGGTGAAGTTGTTGGTTTTATCGGACCAAATGGA 120

WT1402 AAAGATGTCTCATTCACAGTAAACGCCGGTGAAGTTGTTGGTTTTATCGGACCAAATGGA 120

WT1190 AAAGATGTCTCATTCACAGTAAACGCCGGTGAAGTTGTTGGTTTTATCGGACCAAATGGA 120

WT1251 AAAGATGTCTCATTCACAGTAAACGCCGGTGAAGTTGTTGGTTTTATCGGACCAAATGGA 120

************************************************************

WT1343 GCAGGAAAGTCAACGACGATCCGTACACTGCTAGGAATCATCAACCGAGACGAAGGAGAT 180

WT1301 GCAGGAAAGTCAACGACGATTCGTACACTGCTAGGAATCATCAACCGAGACGAAGGAGAT 180

WT1145 GCAGGAAAGTCAACGACGATTCGTACACTGCTAGGAATCATCAACCGAGACGAAGGAGAT 180

WT2608 GCAGGAAAGTCAACGACGATTCGTACACTGCTAGGAATCATCAACCGAGACGAAGGAGAT 180

WT1402 GCAGGAAAGTCAACGACGATTCGTACACTGCTAGGAATCATCAACCGAGACGAAGGAGAT 180

WT1190 GCAGGAAAGTCAACGACGATTCGTACACTGCTAGGAATCATCAACCGAGACGAAGGAGAT 180

WT1251 GCAGGAAAGTCAACGACGATTCGTACACTGCTAGGAATCATCAACCGAGACGAAGGAGAT 180

******************** ***************************************

WT1343 GTCCAAATATTCGGAAAAGATGTTTGGAAAGATAGTCTAGAAATCCATAAACGAATTTCG 240

WT1301 GTCCAAATATTCGGAAAAGATGTTTGGAAAGATAGTCTAGAAATCCATAAACGAATTTCG 240

WT1145 GTCCAAATATTCGGAAAAGATGTTTGGAAAGATAGTCTAGAAATCCATAAACGAATTTCG 240

WT2608 GTCCAAATATTCGGAAAAGATGTTTGGAAAGATAGTCTAGAAATCCATAAACGAATTTCG 240

WT1402 GTCCAAATATTCGGAAAAGATGTTTGGAAAGATAGTCTAGAAATCCATAAACGAATTTCG 240

WT1190 GTCCAAATATTCGGAAAAGATGTTTGGAAAGATAGTCTAGAAATCCATAAACGAATTTCG 240

WT1251 GTCCAAATATTCGGAAAAGATGTTTGGAAAGATAGTCTAGAAATCCATAAACGAATTTCG 240

************************************************************

WT1343 TATGTTCCTGGAGATGTTGCTCTTTGGGGCAGCCTGACTGGTGGAGAGATCATTGATCTA 300

WT1301 TATGTTCCTGGGGATGTTGCTCTTTGGGGCAGCCTGACTGGTGGAGAGATCATTGATCTA 300

WT1145 TATGTTCCTGGGGATGTTGCTCTTTGGGGCAGCCTGACTGGTGGAGAGATCATTGATCTA 300

WT2608 TATGTTCCTGGGGATGTTGCTCTTTGGGGCAGCCTGACTGGTGGAGAGATCATTGATCTA 300

WT1402 TATGTTCCTGGGGATGTTGCTCTTTGGGGCAGCCTGACTGGTGGAGAGATCATTGATCTA 300

WT1190 TATGTTCCTGGGGATGTTGCTCTTTGGGGCAGCCTGACTGGTGGAGAGATCATTGATCTA 300

WT1251 TATGTTCCTGGGGATGTTGCTCTTTGGGGCAGCCTGACTGGTGGAGAGATCATTGATCTA 300

*********** ************************************************

WT1343 TTTATCAAACTTCATGGCGGCGGGAGCAAAGCAAAGCGCGATTATTTAATCAAACGATTT 360

WT1301 TTTATCAAACTTCATGGCGGCGGGAGCAAAGCAAAGCGTGATTATTTAATCAAACGATTT 360

WT1145 TTTATCAAACTTCATGGCGGCGGGAGCAAAGCAAAGCGTGATTATTTAATCAAACGATTT 360

WT2608 TTTATCAAACTTCATGGCGGCGGGAGCAAAGCAAAGCGTGATTATTTAATCAAACGATTT 360

WT1402 TTTATCAAACTTCATGGCGGCGGGAGCAAAGCAAAGCGTGATTATTTAATCAAACGATTT 360

WT1190 TTTATCAAACTTCATGGCGGCGGGAGCAAAGCAAAGCGTGATTATTTAATCAAACGATTT 360

WT1251 TTTATCAAACTTCATGGCGGCGGGAGCAAAGCAAAGCGTGATTATTTAATCAAACGATTT 360

************************************** *********************

WT1343 GAACTTGATCCAAAGAAAAAAGCCAAAGGTTACTCTAAAGGAAATCGTCAAAAAGTCGGT 420

WT1301 GAACTTGATCCAAAGAAAAAAGCCAAAGGTTACTCTAAAGGAAATCGTCAAAAAGTCGGT 420

WT1145 GAACTTGATCCAAAGAAAAAAGCCAAAGGTTACTCTAAAGGAAATCGTCAAAAAGTCGGT 420

WT2608 GAACTTGATCCAAAGAAAAAAGCCAAAGGTTACTCTAAAGGAAATCGTCAAAAAGTCGGT 420

WT1402 GAACTTGATCCAAAGAAAAAAGCCAAAGGTTACTCTAAAGGAAATCGTCAAAAAGTCGGT 420

WT1190 GAACTTGATCCAAAGAAAAAAGCCAAAGGTTACTCTAAAGGAAATCGTCAAAAAGTCGGT 420

WT1251 GAACTTGATCCAAAGAAAAAAGCCAAAGGTTACTCTAAAGGAAATCGTCAAAAAGTCGGT 420

************************************************************

WT1343 TTGATTGCTGCACTTTCAGTTGAATCTGATCTGTATATTTTAGATGAACCGACTTCAGGA 480

WT1301 TTGATTGCTGCACTTTCAGTTGAATCTGATCTGTATATTTTAGATGAACCGACTTCAGGA 480

WT1145 TTGATTGCTGCACTTTCAGTTGAATCTGATCTGTATATTTTAGATGAACCGACTTCAGGA 480

WT2608 TTGATTGCTGCACTTTCAGTTGAATCTGATCTGTATATTTTAGATGAACCGACTTCAGGA 480

WT1402 TTGATTGCTGCACTTTCAGTTGAATCTGATCTGTATATTTTAGATGAACCGACTTCAGGA 480

WT1190 TTGATTGCTGCACTTTCAGTTGAATCTGATCTGTATATTTTAGATGAACCGACTTCAGGA 480

WT1251 TTGATTGCTGCACTTTCAGTTGAATCTGATCTGTATATTTTAGATGAACCGACTTCAGGA 480

************************************************************

WT1343 CTAGATCCATTGATGGAAGCAGTATTCCAAGAAGAAGTAGAAAAAATCAAAAATGATGGC 540

WT1301 CTAGATCCATTGATGGAAGCAGTATTCCAAGAAGAAGTAGAAAAAATCAAAAATGATGGC 540

WT1145 CTAGATCCATTGATGGAAGCAGTATTCCAAGAAGAAGTAGAAAAAATCAAAAATGATGGC 540

WT2608 CTAGATCCATTGATGGAAGCAGTATTCCAAGAAGAAGTAGAAAAAATCAAAAATGATGGC 540

WT1402 CTAGATCCATTGATGGAAGCAGTATTCCAAGAAGAAGTAGAAAAAATCAAAAATGATGGC 540

WT1190 CTAGATCCATTGATGGAAGCAGTATTCCAAGAAGAAGTAGAAAAAATCAAAAATGATGGC 540

WT1251 CTAGATCCATTGATGGAAGCAGTATTCCAAGAAGAAGTAGAAAAAATCAAAAATGATGGC 540

************************************************************

WT1343 AAAGCGATTCTATTATCTTCACATATTTTAAGTGAAGTTGAACGATTAGCAGATAAAGTA 600

WT1301 AAAGCGATTCTATTATCTTCACATATTTTAAGTGAAGTTGAACGATTAGCAGATAAAGTA 600

WT1145 AAAGCGATTCTATTATCTTCACATATTTTAAGTGAAGTTGAACGATTAGCAGATAAAGTA 600

WT2608 AAAGCGATTCTATTATCTTCACATATTTTAAGTGAAGTTGAACGATTAGCAGATAAAGTA 600

WT1402 AAAGCGATTCTATTATCTTCACATATTTTAAGTGAAGTTGAACGATTAGCAGATAAAGTA 600

WT1190 AAAGCGATTCTATTATCTTCACATATTTTAAGTGAAGTTGAACGATTAGCAGATAAAGTA 600

WT1251 AAAGCGATTCTATTATCTTCACATATTTTAAGTGAAGTTGAACGATTAGCAGATAAAGTA 600

************************************************************

WT1343 GCAATCATTCGACGTGGAGAAGTAGTTGAAACAGGTACATTAGATGAATTGCGTCATTTG 660

WT1301 GCAATCATTCGACGTGGAGAAGTAGTTGAAACAGGTACATTAGATGAATTGCGTCATTTG 660

WT1145 GCAATCATTCGACGTGGAGAAGTAGTTGAAACAGGTACATTAGATGAATTGCGTCATTTG 660

WT2608 GCAATCATTCGACGTGGAGAAGTAGTTGAAACAGGTACATTAGATGAATTGCGTCATTTG 660

WT1402 GCAATCATTCGACGTGGAGAAGTAGTTGAAACAGGTACATTAGATGAATTGCGTCATTTG 660

WT1190 GCAATCATTCGACGTGGAGAAGTAGTTGAAACAGGTACATTAGATGAATTGCGTCATTTG 660

WT1251 GCAATCATTCGACGTGGAGAAGTAGTTGAAACAGGTACATTAGATGAATTGCGTCATTTG 660

************************************************************

WT1343 ACTCGCTCAACAGTTACATTGGTGACAAAAGGCGATATTGAGAAACTTGCGACGCTCTCT 720

WT1301 ACTCGCTCAACAGTTACATTGGTGACAAAAGGCGATATTGAGAAACTTGCGACGCTCTCT 720

WT1145 ACTCGCTCAACAGTTACATTGGTGACAAAAGGCGATATTGAGAAACTTGCGACGCTCTCT 720

WT2608 ACTCGCTCAACAGTTACATTGGTGACAAAAGGCGATATTGAGAAACTTGCGACGCTCTCT 720

WT1402 ACTCGCTCAACAGTTACATTGGTGACAAAAGGCGATATTGAGAAACTTGCGACGCTCTCT 720

WT1190 ACTCGCTCAACAGTTACATTGGTGACAAAAGGCGATATTGAGAAACTTGCGACGCTCTCT 720

WT1251 ACTCGCTCAACAGTTACATTGGTGACAAAAGGCGATATTGAGAAACTTGCGACGCTCTCT 720

************************************************************

WT1343 GGCGTGCATGATTTTGTTCAAAAAGACGGCAAAGCGACTTTTTCTGCTGACAATGAAGCG 780

WT1301 GGCGTGCATGATTTTGTTCAAAAAGACGGCAAAGCAACTTTTTCTGCTGACAATGAAGCG 780

WT1145 GGCGTGCATGATTTTGTTCAAAAAGACGGCAAAGCAACTTTTTCTGCTGACAATGAAGCG 780

WT2608 GGCGTGCATGATTTTGTTCAAAAAGACGGCAAAGCAACTTTTTCTGCTGACAATGAAGCG 780

WT1402 GGCGTGCATGATTTTGTTCAAAAAGACGGCAAAGCAACTTTTTCTGCTGACAATGAAGCG 780

WT1190 GGCGTGCATGATTTTGTTCAAAAAGACGGCAAAGCAACTTTTTCTGCTGACAATGAAGCG 780

WT1251 GGCGTGCATGATTTTGTTCAAAAAGACGGCAAAGCAACTTTTTCTGCTGACAATGAAGCG 780

*********************************** ************************

WT1343 ATAAATACGATTCTGACCGAGGCAACCAAATTAGGTGTGACAAAAATCGAATCTGTACCG 840

WT1301 ATGAATACGATTCTGACCGAGGCAACCAAATTAGGTGTGATAAAAATCGAATCTGTACCG 840

WT1145 ATGAATACGATTCTGACCGAGGCAACCAAATTAGGTGTGATAAAAATCGAATCTGTACCG 840

WT2608 ATGAATACGATTCTGACCGAGGCAACCAAATTAGGTGTGATAAAAATCGAATCTGTACCG 840

WT1402 ATGAATACGATTCTGACCGAGGCAACCAAATTAGGTGTGATAAAAATCGAATCTGTACCG 840

WT1190 ATGAATACGATTCTGACCGAGGCAACCAAATTAGGTGTGATAAAAATCGAATCTGTACCG 840

WT1251 ATGAATACGATTCTGACCGAGGCAACCAAATTAGGTGTGATAAAAATCGAATCTGTACCG 840

** ************************************* *******************

WT1343 CCAACGCTTGAAGATTTATTCATGCGTCACTACGAAGGCTGATTGTCGGAACGGAGGAAA 900

WT1301 CCAACGCTCGAAGATTTATTCATGCGTCACTACGAAGGCTGATTGTCGGAACGGAGGAAA 900

WT1145 CCAACGCTCGAAGATTTATTCATGCGTCACTACGAAGGCTGATTGTCGGAACGGAGGAAA 900

WT2608 CCAACGCTCGAAGATTTATTCATGCGTCACTACGAAGGCTGATTGTCGGAACGGAGGAAA 900

WT1402 CCAACGCTCGAAGATTTATTCATGCGTCACTACGAAGGCTGATTGTCGGAACGGAGGAAA 900

WT1190 CCAACGCTCGAAGATTTATTCATGCGTCACTACGAAGGCTGATTGTCGGAACGGAGGAAA 900

WT1251 CCAACGCTCGAAGATTTATTCATGCGTCACTACGAAGGCTGATTGTCGGAACGGAGGAAA 900

******** ***************************************************

WT1343 AGAAAATGAATGAAAAATTTGCCCGTTGGAACGTATTGTTCATTCAATACGTGAAACGCG 960

WT1301 AGAAAATGAATGAAAAATTTGCGCGTTGGAACGTATTGTTCATTCAATACGTGAAACGCG 960

WT1145 AGAAAATGAATGAAAAATTTGCGCGTTGGAACGTATTGTTCATTCAATACGTGAAACGCG 960

WT2608 AGAAAATGAATGAAAAATTTGCGCGTTGGAACGTATTGTTCATTCAATACGTGAAACGCG 960

WT1402 AGAAAATGAATGAAAAATTTGCGCGTTGGAACGTATTGTTCATTCAATACGTGAAACGCG 960

WT1190 AGAAAATGAATGAAAAATTTGCGCGTTGGAACGTATTGTTCATTCAATACGTGAAACGCG 960

WT1251 AGAAAATGAATGAAAAATTTGCGCGTTGGAACGTATTGTTCATTCAATACGTGAAACGCG 960

********************** *************************************

WT1343 ATTGGAAAAAAATAATTGTTTGGGTTTTAGGTTTGGGTTTGTTCTCAGGAGCATTTGTAC 1020

WT1301 ATTGGAAAAAAATAATTGTTTGGGTTTTAGGTTTGGGTTTGTTCTCAGGAGCATTTGTAC 1020

WT1145 ATTGGAAAAAAATAATTGTTTGGGTTTTAGGTTTGGGTTTGTTCTCAGGAGCATATGTAC 1020

WT2608 ATTGGAAAAAAATAATTGTTTGGGTTTTAGGTTTGGGTTTGTTCTCAGGAGCATATGTAC 1020

WT1402 ATTGGAAAAAAATAATTGTTTGGGTTTTAGGTTTGGGTTTGTTCTCAGGAGCATATGTAC 1020

WT1190 ATTGGAAAAAAATAATTGTTTGGGTTTTAGGTTTGGGTTTGTTCTCAGGAGCATTTGTAC 1020

WT1251 ATTGGAAAAAAATAATTGTTTGGGTTTTAGGTTTGGGTTTGTTCTCAGGAGCATTTGTAC 1020

****************************************************** *****

WT1343 CAGCATTTGAAGAGATTGCTAAAGGACAAGGTCTTTTAGGGATGTTTGAAACGATGCAAA 1080

WT1301 CAGCATTTGAAGAGATTGCTAAAGGACAAGGTCTTTTAGGGATGTTTGAAACGATGCAAA 1080

WT1145 CAGCATTTGAAGAGATTGCTAAAGGACAAGGTCTTTTAGGGATGTTTGAAACGATGCAAA 1080

WT2608 CAGCATTTGAAGAGATTGCTAAAGGACAAGGTCTTTTAGGGATGTTTGAAACGATGCAAA 1080

WT1402 CAGCATTTGAAGAGATTGCTAAAGGACAAGGTCTTTTAGGGATGTTTGAAACGATGCAAA 1080

WT1190 CAGCATTTGAAGAGATTGCTAAAGGACAAGGTCTTTTAGGGATGTTTGAAACGATGCAAA 1080

WT1251 CAGCATTTGAAGAGATTGCTAAAGGACAAGGTCTTTTAGGGATGTTTGAAACGATGCAAA 1080

************************************************************

WT1343 ATCCAGCGATGATCTCGATGGTTGGACCTACACCAATCAAAATAGGTACGGATTATACTT 1140

WT1301 ATCCAGCGATGATCTCGATGGTTGGACCTACACCAATCAAAATAGGTACGGATTATACTT 1140

WT1145 ATCCAGCGATGATCTCGATGGTTGGACCTACACCAATCAAAATAGGTACGGATTATACTT 1140

WT2608 ATCCAGCGATGATCTCGATGGTTGGACCTACACCAATCAAAATAGGTACGGATTATACTT 1140

WT1402 ATCCAGCGATGATCTCGATGGTTGGACCTACACCAATCAAAATAGGTACGGATTATACTT 1140

WT1190 ATCCAGCGATGATCTCGATGGTTGGACCTACACCAATCAAAATAGGTACGGATTATACTT 1140

WT1251 ATCCAGCGATGATCTCGATGGTTGGACCTACACCAATCAAAATAGGTACGGATTATACTT 1140

************************************************************

WT1343 TAGGAGCGATGTATGCTCAAGAGATGTTGCTGTTTTGCGGATTGTTCGCAATGATTATCT 1200

WT1301 TAGGAGCGATGTATGCTCAAGAGATGTTGCTGTTTTGCGGATTGTTCGCAATGATTATCT 1200

WT1145 TAGGAGCGATGTATGCTCAAGAGATGTTGCTGTTTTGCGGATTGTTCGCAATGATTATCT 1200

WT2608 TAGGAGCGATGTATGCTCAAGAGATGTTGCTGTTTTGCGGATTGTTCGCAATGATTATCT 1200

WT1402 TAGGAGCGATGTATGCTCAAGAGATGTTGCTGTTTTGCGGATTGTTCGCAATGATTATCT 1200

WT1190 TAGGAGCGATGTATGCTCAAGAGATGTTGCTGTTTTGCGGATTGTTCGCAATGATTATCT 1200

WT1251 TAGGAGCGATGTATGCTCAAGAGATGTTGCTGTTTTGCGGATTGTTCGCAATGATTATCT 1200

************************************************************

WT1343 CAGCACTTCATGTGGTGAGCCACACGCGAAAAGAAGAAGAATTAGGTTTGACTGAATTGG 1260

WT1301 CAGCACTTCATGTGGTGAGCCACACGCGAAAAGAAGAAGAATTAGGTTTGACTGAATTGG 1260

WT1145 CAGCACTTCATGTGGTGAGCCACACGCGAAAAGAAGAAGAATTAGGTTTGACTGAATTGG 1260

WT2608 CAGCACTTCATGTGGTGAGCCACACGCGAAAAGAAGAAGAATTAGGTTTGACTGAATTGG 1260

WT1402 CAGCACTTCATGTGGTGAGCCACACGCGAAAAGAAGAAGAATTAGGTTTGACTGAATTGG 1260

WT1190 CAGCACTTCATGTGGTGAGCCACACGCGAAAAGAAGAAGAATTAGGTTTGACTGAATTGG 1260

WT1251 CAGCACTTCATGTGGTGAGCCACACGCGAAAAGAAGAAGAATTAGGTTTGACTGAATTGG 1260

************************************************************

WT1343 TTCGCTCATTTCGAGTAGGACGACAAGCCAATTCATTAGCTGTTATCAGTGAGATGCTGT 1320

WT1301 TTCGCTCATTTCGAGTAGGACGACAAGCCAATTCATTAGCTGTTATCAGTGAGATGCTGT 1320

WT1145 TTCGCTCATTTCGAGTAGGACGACAAGCCAATTCATTAGCTGTTATCAGTGAGATGCTGT 1320

WT2608 TTCGCTCATTTCGAGTAGGACGACAAGCCAATTCATTAGCTGTTATCAGTGAGATGCTGT 1320

WT1402 TTCGCTCATTTCGAGTAGGACGACAAGCCAATTCATTAGCTGTTATCAGTGAGATGCTGT 1320

WT1190 TTCGCTCATTTCGAGTAGGACGACAAGCCAATTCATTAGCTGTTATCAGTGAGATGCTGT 1320

WT1251 TTCGCTCATTTCGAGTAGGACGACAAGCCAATTCATTAGCTGTTATCAGTGAGATGCTGT 1320

************************************************************

WT1343 TGATCAATCTTTTATTAGGTCTTTTAATCGGCGGACTCATGATGAGTTTTGGTGTAAAAA 1380

WT1301 TGATCAATCTTTTATTAGGTCTTTTAATCGGCGGACTCATGATGAGTTTTGGTGTAAAAA 1380

WT1145 TGATCAATCTTTTATTAGGTCTTTTAATCGGCGGACTCATGATGAGTTTTGGTGTAAAAA 1380

WT2608 TGATCAATCTTTTATTAGGTCTTTTAATCGGCGGACTCATGATGAGTTTTGGTGTAAAAA 1380

WT1402 TGATCAATCTTTTATTAGGTCTTTTAATCGGCGGACTCATGATGAGTTTTGGTGTAAAAA 1380

WT1190 TGATCAATCTTTTATTAGGTCTTTTAATCGGCGGACTCATGATGAGTTTTGGTGTAAAAA 1380

WT1251 TGATCAATCTTTTATTAGGTCTTTTAATCGGCGGACTCATGATGAGTTTTGGTGTAAAAA 1380

************************************************************

WT1343 CGATTGATGCCGAAGGAGCTTTCTTGTTCGGAGGATCAATTGCATTGGCGGGAATTATCG 1440

WT1301 CGATTGATGCCGAAGGAGCTTTCTTGTTCGGAGGATCAATTGCATTGGCGGGAATTATCG 1440

WT1145 CGATTGATGCCGAAGGAGCTTTCTTGTTCGGAGGATCAATTGCATTGGCGGGAATTATCG 1440

WT2608 CGATTGATGCCGAAGGAGCTTTCTTGTTCGGAGGATCAATTGCATTGGCGGGAATTATCG 1440

WT1402 CGATTGATGCCGAAGGAGCTTTCTTGTTCGGAGGATCAATTGCATTGGCGGGAATTATCG 1440

WT1190 CGATTGATGCCGAAGGAGCTTTCTTGTTCGGAGGATCAATTGCATTGGCGGGAATTATCG 1440

WT1251 CGATTGATGCCGAAGGAGCTTTCTTGTTCGGAGGATCAATTGCATTGGCGGGAATTATCG 1440

************************************************************

WT1343 GTGGTGTATTGGCACTTGTGATGTCGCAGATTATGGCGACTTCTACTGGAGCAACCGGCT 1500

WT1301 GTGGTGTATTGGCACTTGTGATGTCGCAGATTATGGCGACTTCTACTGGAGCAACCGGCT 1500

WT1145 GTGGTGTATTGGCACTTGTGATGTCGCAGATTATGGCGACTTCTACTGGAGCAACCGGCT 1500

WT2608 GTGGTGTATTGGCACTTGTGATGTCGCAGATTATGGCGACTTCTACTGGAGCAACCGGCT 1500

WT1402 GTGGTGTATTGGCACTTGTGATGTCGCAGATTATGGCGACTTCTACTGGAGCAACCGGCT 1500

WT1190 GTGGTGTATTGGCACTTGTGATGTCGCAGATTATGGCGACTTCTACTGGAGCAACCGGCT 1500

WT1251 GTGGTGTATTGGCACTTGTGATGTCGCAGATTATGGCGACTTCTACTGGAGCAACCGGCT 1500

************************************************************

WT1343 CGACATTAAGTCTTATAGGACTTTTGTATATCGTGCGCGCTGGAACAGATGTGTCTAATC 1560

WT1301 CGACATTAAGTCTTATAGGACTTTTGTATATCGTGCGCGCTGGAACAGATGTGTCTAATC 1560

WT1145 CGACATTAAGTCTTATAGGACTTTTGTATATCGTGCGCGCTGGAACAGATGTGTCTAATC 1560

WT2608 CGACATTAAGTCTTATAGGACTTTTGTATATCGTGCGCGCTGGAACAGATGTGTCTAATC 1560

WT1402 CGACATTAAGTCTTATAGGACTTTTGTATATCGTGCGCGCTGGAACAGATGTGTCTAATC 1560

WT1190 CGACATTAAGTCTTATAGGACTTTTGTATATCGTGCGCGCTGGAACAGATGTGTCTAATC 1560

WT1251 CGACATTAAGTCTTATAGGACTTTTGTATATCGTGCGCGCTGGAACAGATGTGTCTAATC 1560

************************************************************

WT1343 TTGATCTATCAATGTTCAATCCAATGGGATGGATTTACTTGACCTATCCTTTCACAAAAA 1620

WT1301 TTGATCTATCAATGTTCAATCCAATGGGATGGATTTACTTGACCTATCCTTTCACAAAAA 1620

WT1145 TTGATCTATCAATGTTCAATCCAATGGGATGGATTTACTTGACCTATCCTTTCACAAAAA 1620

WT2608 TTGATCTATCAATGTTCAATCCAATGGGATGGATTTACTTGACCTATCCTTTCACAAAAA 1620

WT1402 TTGATCTATCAATGTTCAATCCAATGGGATGGATTTACTTGACCTATCCTTTCACAAAAA 1620

WT1190 TTGATCTATCAATGTTCAATCCAATGGGATGGATTTACTTGACCTATCCTTTCACAAAAA 1620

WT1251 TTGATCTATCAATGTTCAATCCAATGGGATGGATTTACTTGACCTATCCTTTCACAAAAA 1620

************************************************************

WT1343 ATAACTGGCTACCATTATTATTTGCTTTGATTTTTAGTCTTGTTTTTACCGTACTTGCGT 1680

WT1301 ATAACTGGCTACCATTATTATTTGCTTTGATTTTTAGTCTTGTTTTTACCGTACTTGCGT 1680

WT1145 ATAACTGGCTACCATTATTATTTGCTTTGATTTTTAGTCTTGTTTTTACCGTACTTGCGT 1680

WT2608 ATAACTGGCTACCATTATTATTTGCTTTGATTTTTAGTCTTGTTTTTACCGTACTTGCGT 1680

WT1402 ATAACTGGCTACCATTATTATTTGCTTTGATTTTTAGTCTTGTTTTTACCGTACTTGCGT 1680

WT1190 ATAACTGGCTACCATTATTATTTGCTTTGATTTTTAGTCTTGTTTTTACCGTACTTGCGT 1680

WT1251 ATAACTGGCTACCATTATTATTTGCTTTGATTTTTAGTCTTGTTTTTACCGTACTTGCGT 1680

************************************************************

WT1343 TTGTGTTGGAAGAACATCGCGACATGGGCGCAGGTTATCTTCCTGAACGAGAAGGACGTG 1740

WT1301 TTGTGTTGGAAGAACATCGCGACATGGGCGCAGGTTATCTTCCTGAACGAGAAGGACGTG 1740

WT1145 TTGTGTTGGAAGAACATCGCGACATGGGCGCAGGTTATCTTCCTGAACGAGAAGGACGTG 1740

WT2608 TTGTGTTGGAAGAACATCGCGACATGGGCGCAGGTTATCTTCCTGAACGAGAAGGACGTG 1740

WT1402 TTGTGTTGGAAGAACATCGCGACATGGGCGCAGGTTATCTTCCTGAACGAGAAGGACGTG 1740

WT1190 TTGTGTTGGAAGAACATCGCGACATGGGCGCAGGTTATCTTCCTGAACGAGAAGGACGTG 1740

WT1251 TTGTGTTGGAAGAACATCGCGACATGGGCGCAGGTTATCTTCCTGAACGAGAAGGACGTG 1740

************************************************************

WT1343 CGACGGCGAAGAAATCACTACTTTCTGTACCTGGTTTGTTTTTCAAGATTAATAAAGGAG 1800

WT1301 CGACGGCGAAGAAATCACTACTTTCTGTACCTGGTTTGTTTTTCAAGATTAATAAAGGAG 1800

WT1145 CGACGGCGAAGAAATCACTACTTTCTGTACCTGGTTTGTTTTTCAAGATTAATAAAGGAG 1800

WT2608 CGACGGCGAAGAAATCACTACTTTCTGTACCTGGTTTGTTTTTCAAGATTAATAAAGGAG 1800

WT1402 CGACGGCGAAGAAATCACTACTTTCTGTACCTGGTTTGTTTTTCAAGATTAATAAAGGAG 1800

WT1190 CGACGGCGAAGAAATCACTACTTTCTGTACCTGGTTTGTTTTTCAAGATTAATAAAGGAG 1800

WT1251 CGACGGCGAAGAAATCACTACTTTCTGTACCTGGTTTGTTTTTCAAGATTAATAAAGGAG 1800

************************************************************

WT1343 TAATGATTGGTTGGCTGATCGCATTTGTGGTTATGGGAGCTGCGTATGGCTCCATTTATG 1860

WT1301 TAATGATTGGTTGGCTGATCGCATTTGTGGTTATGGGAGCTGCGTATGGCTCCATTTATG 1860

WT1145 TAATGATTGGTTGGCTGATCGCATTTGTGGTTATGGGAGCTGCGTATGGCTCCATTTATG 1860

WT2608 TAATGATTGGTTGGCTGATCGCATTTGTGGTTATGGGAGCTGCGTATGGCTCCATTTATG 1860

WT1402 TAATGATTGGTTGGCTGATCGCATTTGTGGTTATGGGAGCTGCGTATGGCTCCATTTATG 1860

WT1190 TAATGATTGGTTGGCTGATCGCATTTGTGGTTATGGGAGCTGCGTATGGCTCCATTTATG 1860

WT1251 TAATGATTGGTTGGCTGATCGCATTTGTGGTTATGGGAGCTGCGTATGGCTCCATTTATG 1860

************************************************************

WT1343 GAGACATGCAAGTCTTTCTTGGCGGAAATGAACTGATGAAACAAATGTTCACTCAATCTG 1920

WT1301 GAGACATGCAAGTCTTTCTTGGCGGAAATGAACTGATGAAACAAATGTTCACTCAATCTG 1920

WT1145 GAGACATGCAAGTCTTTCTTGGCGGAAATGAACTGATGAAACAAATGTTCACTCAATCTG 1920

WT2608 GAGACATGCAAGTCTTTCTTGGCGGAAATGAACTGATGAAACAAATGTTCACTCAATCTG 1920

WT1402 GAGACATGCAAGTCTTTCTTGGCGGAAATGAACTGATGAAACAAATGTTCACTCAATCTG 1920

WT1190 GAGACATGCAAGTCTTTCTTGGCGGAAATGAACTGATGAAACAAATGTTCACTCAATCTG 1920

WT1251 GAGACATGCAAGTCTTTCTTGGCGGAAATGAACTGATGAAACAAATGTTCACTCAATCTG 1920

************************************************************

WT1343 GCGTTTCCATTGAAGAATCCTTTACGGCAACGATCATGATGGTAATGATTGGATTAGTCA 1980

WT1301 GCGTTTCCATTGAAGAATCCTTTACGGCAACGATCATGATGGTAATGATTGGATTAGTCA 1980

WT1145 GCGTTTCCATTGAAGAATCCTTTACGGCAACGATCATGATGGTAATGATTGGATTAGTCA 1980

WT2608 GCGTTTCCATTGAAGAATCCTTTACGGCAACGATCATGATGGTAATGATTGGATTAGTCA 1980

WT1402 GCGTTTCCATTGAAGAATCCTTTACGGCAACGATCATGATGGTAATGATTGGATTAGTCA 1980

WT1190 GCGTTTCCATTGAAGAATCCTTTACGGCAACGATCATGATGGTAATGATTGGATTAGTCA 1980

WT1251 GCGTTTCCATTGAAGAATCCTTTACGGCAACGATCATGATGGTAATGATTGGATTAGTCA 1980

************************************************************

WT1343 CAATCTTGCCAATCGCGGTGGTCAATAAATTATTTGCAGAAGAAACAAGACTGCATCTGA 2040

WT1301 CAATCTTGCCAATCGCGGTGGTCAATAAATTATTTGCAGAAGAAACAAGACTGCATCTGA 2040

WT1145 CAATCTTGCCAATCGCGGTGGTCAATAAATTATTTGCAGAAGAAACAAGACTGCATCTGA 2040

WT2608 CAATCTTGCCAATCGCGGTGGTCAATAAATTATTTGCAGAAGAAACAAGACTGCATCTGA 2040

WT1402 CAATCTTGCCAATCGCGGTGGTCAATAAATTATTTGCAGAAGAAACAAGACTGCATCTGA 2040

WT1190 CAATCTTGCCAATCGCGGTGGTCAATAAATTATTTGCAGAAGAAACAAGACTGCATCTGA 2040

WT1251 CAATCTTGCCAATCGCGGTGGTCAATAAATTATTTGCAGAAGAAACAAGACTGCATCTGA 2040

************************************************************

WT1343 GTCAACTGTATGTAACGAAGATTACGCGAGGCCAATTATATTGGACAACGATATTTTTAG 2100

WT1301 GTCAACTGTATGTAACGAAGATTACGCGAGGCCAATTATATTGGACAACGATATTTTTAG 2100

WT1145 GTCAACTGTATGTAACGAAGATTACGCGAGGCCAATTATATTGGACAACGATATTTTTAG 2100

WT2608 GTCAACTGTATGTAACGAAGATTACGCGAGGCCAATTATATTGGACAACGATATTTTTAG 2100

WT1402 GTCAACTGTATGTAACGAAGATTACGCGAGGCCAATTATATTGGACAACGATATTTTTAG 2100

WT1190 GTCAACTGTATGTAACGAAGATTACGCGAGGCCAATTATATTGGACAACGATATTTTTAG 2100

WT1251 GTCAACTGTATGTAACGAAGATTACGCGAGGCCAATTATATTGGACAACGATATTTTTAG 2100

************************************************************

WT1343 CTATTTTTGCTGGAGTCGTAGGCATTGGCTTAGCATCAGCGGGCTTAGGTGGAACGGCGA 2160

WT1301 CTATTTTTGCTGGAGTCGTAGGCATTGGCTTAGCATCAGCGGGATTAGGTGGAACGGCGA 2160

WT1145 CTATTTTTGCTGGAGTCGTAGGCATTGGCTTAGCATCAGCGGGATTAGGTGGAACGGCGA 2160

WT2608 CTATTTTTGCTGGAGTCGTAGGCATTGGCTTAGCATCAGCGGGATTAGGTGGAACGGCGA 2160

WT1402 CTATTTTTGCTGGAGTCGTAGGCATTGGCTTAGCATCAGCGGGATTAGGTGGAACGGCGA 2160

WT1190 CTATTTTTGCTGGAGTCGTAGGCATTGGCTTAGCATCAGCGGGATTAGGTGGAACGGCGA 2160

WT1251 CTATTTTTGCTGGAGTCGTAGGCATTGGCTTAGCATCAGCGGGATTAGGTGGAACGGCGA 2160

******************************************* ****************

WT1343 TTTCTGCGATGAAAAATGAATCGACTATGGATCTGACCGATTTCTTAGCTGCTGGATACA 2220

WT1301 TTTCTGCGATGAAAAATGAATCGACTATGGATCTGACCGATTTCTTAGCTGCTGGATACA 2220

WT1145 TTTCTGCGATGAAAAATGAATCGACTATGGATCTGACCGATTTCTTAGCTGCTGGATACA 2220

WT2608 TTTCTGCGATGAAAAATGAATCGACTATGGATCTGACCGATTTCTTAGCTGCTGGATACA 2220

WT1402 TTTCTGCGATGAAAAATGAATCGACTATGGATCTGACCGATTTCTTAGCTGCTGGATACA 2220

WT1190 TTTCTGCGATGAAAAATGAATCGACTATGGATCTGACCGATTTCTTAGCTGCTGGATACA 2220

WT1251 TTTCTGCGATGAAAAATGAATCGACTATGGATCTGACCGATTTCTTAGCTGCTGGATACA 2220

************************************************************

WT1343 ATTTTCTCCCTTCCATCTTATTTTATATTGGTTTGGCTGCTTTAGCGTTAGGCTGGTTGC 2280

WT1301 ATTTTCTCCCTTCCATCTTATTTTATATTGGTTTGGCTGCTTTAGCGTTAGGCTGGTTGC 2280

WT1145 ATTTTCTCCCTTCCATCTTATTTTATATTGGTTTGGCTGCTTTAGCGTTAGGCTGGTTGC 2280

WT2608 ATTTTCTCCCTTCCATCTTATTTTATATTGGTTTGGCTGCTTTAGCGTTAGGCTGGTTGC 2280

WT1402 ATTTTCTCCCTTCCATCTTATTTTATATTGGTTTGGCTGCTTTAGCGTTAGGCTGGTTGC 2280

WT1190 ATTTTCTCCCTTCCATCTTATTTTATATTGGTTTGGCTGCTTTAGCGTTAGGCTGGTTGC 2280

WT1251 ATTTTCTCCCTTCCATCTTATTTTATATTGGTTTGGCTGCTTTAGCGTTAGGCTGGTTGC 2280

************************************************************

WT1343 CAAAATTTGGAAAAGTAATCTATGCTTATCTAGGCTATTCCTTTGCTTTGAATTATTTCG 2340

WT1301 CAAAATTTGGAAAAGTAATCTATGCTTATCTAGGCTATTCCTTTGCTTTGAATTATTTCG 2340

WT1145 CAAAATTTGGAAAAGTAATCTATGCTTATCTAGGCTATTCCTTTGCTTTGAATTATTTCG 2340

WT2608 CAAAATTTGGAAAAGTAATCTATGCTTATCTAGGCTATTCCTTTGCTTTGAATTATTTCG 2340

WT1402 CAAAATTTGGAAAAGTAATCTATGCTTATCTAGGCTATTCCTTTGCTTTGAATTATTTCG 2340

WT1190 CAAAATTTGGAAAAGTAATCTATGCTTATCTAGGCTATTCCTTTGCTTTGAATTATTTCG 2340

WT1251 CAAAATTTGGAAAAGTAATCTATGCTTATCTAGGCTATTCCTTTGCTTTGAATTATTTCG 2340

************************************************************

WT1343 GCGGAATCTTAGATTTGCCGGATTGGTTCTCAAAAACGGCGATTCAAAGCTGGATTCCAC 2400

WT1301 GCGGAATCTTAGATTTGCCGGATTGGTTCTCAAAAACGGCGATTCAAAGTTGGATTCCAC 2400

WT1145 GCGGAATCTTAGATTTGCCGGATTGGTTCTCAAAAACGGCGATTCAAAGTTGGATTCCAC 2400

WT2608 GCGGAATCTTAGATTTGCCGGATTGGTTCTCAAAAACGGCGATTCAAAGTTGGATTCCAC 2400

WT1402 GCGGAATCTTAGATTTGCCGGATTGGTTCTCAAAAACGGCGATTCAAAGTTGGATTCCAC 2400

WT1190 GCGGAATCTTAGATTTGCCGGATTGGTTCTCAAAAACGGCGATTCAAAGTTGGATTCCAC 2400

WT1251 GCGGAATCTTAGATTTGCCGGATTGGTTCTCAAAAACGGCGATTCAAAGTTGGATTCCAC 2400

************************************************* **********

WT1343 GCTTACCGATGGAAGAATTTGATGGAACGATTTTTGCAGTAATTACTGTTATCAGTATCG 2460

WT1301 GCTTACCGATGGAAGAATTTGATGGAACGATTTTTGCAGTAATTACTGTTATCAGTATCG 2460

WT1145 GCTTACCGATGGAAGAATTTGATGGAACGATTTTTGCAGTAATTACTGTTATCAGTATCG 2460

WT2608 GCTTACCGATGGAAGAATTTGATGGAACGATTTTTGCAGTAATTACTGTTATCAGTATCG 2460

WT1402 GCTTACCGATGGAAGAATTTGATGGAACGATTTTTGCAGTAATTACTGTTATCAGTATCG 2460

WT1190 GCTTACCGATGGAAGAATTTGATGGAACGATTTTTGCAGTAATTACTGTTATCAGTATCG 2460

WT1251 GCTTACCGATGGAAGAATTTGATGGAACGATTTTTGCAGTAATTACTGTTATCAGTATCG 2460

************************************************************

WT1343 TCTTCTTATTTGTCGGCTATTTAGGATACAAACGCCGTGATATGGTAGAAGGCGCTTAA 2519

WT1301 TCTTCTTATTTGTCGGCTATTTAGGATACAAACGCCGTGATATGGTAGAAGGCGCTTAA 2519

WT1145 TCTTCTTATTTGTCGGCTATTTAGGATACAAACGCCGTGATATGGTAGAAGGCGCTTAA 2519

WT2608 TCTTCTTATTTGTCGGCTATTTAGGATACAAACGCCGTGATATGGTAGAAGGCGCTTAA 2519

WT1402 TCTTCTTATTTGTCGGCTATTTAGGATACAAACGCCGTGATATGGTAGAAGGCGCTTAA 2519

WT1190 TCTTCTTATTTGTCGGCTATTTAGGATACAAACGCCGTGATATGGTAGAAGGCGCTTAA 2519

WT1251 TCTTCTTATTTGTCGGCTATTTAGGATACAAACGCCGTGATATGGTAGAAGGCGCTTAA 2519

***********************************************************

**Supplementary Figure 2. Alignment of amino acid sequences of NarA**

WT1343_ATPase MTEIVKVQGLQKKFGKFQALKDVSFTVNAGEVVGFIGPNGAGKSTTIRTLLGIINRDEGD 60

WT1145_ATPase MTEIVKVQGLQKKFGKFQALKDVSFTVNAGEVVGFIGPNGAGKSTTIRTLLGIINRDEGD 60

WT1190_ATPase MTEIVKVQGLQKKFGKFQALKDVSFTVNAGEVVGFIGPNGAGKSTTIRTLLGIINRDEGD 60

WT1251_ATPase MTEIVKVQGLQKKFGKFQALKDVSFTVNAGEVVGFIGPNGAGKSTTIRTLLGIINRDEGD 60

WT1402_ATPase MTEIVKVQGLQKKFGKFQALKDVSFTVNAGEVVGFIGPNGAGKSTTIRTLLGIINRDEGD 60

WT2608_ATPase MTEIVKVQGLQKKFGKFQALKDVSFTVNAGEVVGFIGPNGAGKSTTIRTLLGIINRDEGD 60

WT1301_ATPase MTEIVKVPGLQKKFGKFQALKDVSFTVNAGEVVGFIGPNGAGKSTTIRTLLGIINRDEGD 60

******* ****************************************************

WT1343_ATPase VQIFGKDVWKDSLEIHKRISYVPGDVALWGSLTGGEIIDLFIKLHGGGSKAKRDYLIKRF 120

WT1145_ATPase VQIFGKDVWKDSLEIHKRISYVPGDVALWGSLTGGEIIDLFIKLHGGGSKAKRDYLIKRF 120

WT1190_ATPase VQIFGKDVWKDSLEIHKRISYVPGDVALWGSLTGGEIIDLFIKLHGGGSKAKRDYLIKRF 120

WT1251_ATPase VQIFGKDVWKDSLEIHKRISYVPGDVALWGSLTGGEIIDLFIKLHGGGSKAKRDYLIKRF 120

WT1402_ATPase VQIFGKDVWKDSLEIHKRISYVPGDVALWGSLTGGEIIDLFIKLHGGGSKAKRDYLIKRF 120

WT2608_ATPase VQIFGKDVWKDSLEIHKRISYVPGDVALWGSLTGGEIIDLFIKLHGGGSKAKRDYLIKRF 120

WT1301_ATPase VQIFGKDVWKDSLEIHKRISYVPGDVALWGSLTGGEIIDLFIKLHGGGSKAKRDYLIKRF 120

************************************************************

WT1343_ATPase ELDPKKKAKGYSKGNRQKVGLIAALSVESDLYILDEPTSGLDPLMEAVFQEEVEKIKNDG 180

WT1145_ATPase ELDPKKKAKGYSKGNRQKVGLIAALSVESDLYILDEPTSGLDPLMEAVFQEEVEKIKNDG 180

WT1190_ATPase ELDPKKKAKGYSKGNRQKVGLIAALSVESDLYILDEPTSGLDPLMEAVFQEEVEKIKNDG 180

WT1251_ATPase ELDPKKKAKGYSKGNRQKVGLIAALSVESDLYILDEPTSGLDPLMEAVFQEEVEKIKNDG 180

WT1402_ATPase ELDPKKKAKGYSKGNRQKVGLIAALSVESDLYILDEPTSGLDPLMEAVFQEEVEKIKNDG 180

WT2608_ATPase ELDPKKKAKGYSKGNRQKVGLIAALSVESDLYILDEPTSGLDPLMEAVFQEEVEKIKNDG 180

WT1301_ATPase ELDPKKKAKGYSKGNRQKVGLIAALSVESDLYILDEPTSGLDPLMEAVFQEEVEKIKNDG 180

************************************************************

WT1343_ATPase KAILLSSHILSEVERLADKVAIIRRGEVVETGTLDELRHLTRSTVTLVTKGDIEKLATLS 240

WT1145_ATPase KAILLSSHILSEVERLADKVAIIRRGEVVETGTLDELRHLTRSTVTLVTKGDIEKLATLS 240

WT1190_ATPase KAILLSSHILSEVERLADKVAIIRRGEVVETGTLDELRHLTRSTVTLVTKGDIEKLATLS 240

WT1251_ATPase KAILLSSHILSEVERLADKVAIIRRGEVVETGTLDELRHLTRSTVTLVTKGDIEKLATLS 240

WT1402_ATPase KAILLSSHILSEVERLADKVAIIRRGEVVETGTLDELRHLTRSTVTLVTKGDIEKLATLS 240

WT2608_ATPase KAILLSSHILSEVERLADKVAIIRRGEVVETGTLDELRHLTRSTVTLVTKGDIEKLATLS 240

WT1301_ATPase KAILLSSHILSEVERLADKVAIIRRGEVVETGTLDELRHLTRSTVTLVTKGDIEKLATLS 240

************************************************************

WT1343_ATPase GVHDFVQKDGKATFSADNEAINTILTEATKLGVTKIESVPPTLEDLFMRHYEG 293

WT1145_ATPase GVHDFVQKDGKATFSADNEAMNTILTEATKLGVIKIESVPPTLEDLFMRHYEG 293

WT1190_ATPase GVHDFVQKDGKATFSADNEAMNTILTEATKLGVIKIESVPPTLEDLFMRHYEG 293

WT1251_ATPase GVHDFVQKDGKATFSADNEAMNTILTEATKLGVIKIESVPPTLEDLFMRHYEG 293

WT1402_ATPase GVHDFVQKDGKATFSADNEAMNTILTEATKLGVIKIESVPPTLEDLFMRHYEG 293

WT2608_ATPase GVHDFVQKDGKATFSADNEAMNTILTEATKLGVIKIESVPPTLEDLFMRHYEG 293

WT1301_ATPase GVHDFVQKDGKATFSADNEAMNTILTEATKLGVIKIESVPPTLEDLFMRHYEG 293

********************:************ *******************

**Supplementary Figure 3. Alignment of amino acid sequences of NarB**

WT1145_permease MNEKFARWNVLFIQYVKRDWKKIIVWVLGLGLFSGAYVPAFEEIAKGQGLLGMFETMQNP 60

WT1402_permease MNEKFARWNVLFIQYVKRDWKKIIVWVLGLGLFSGAYVPAFEEIAKGQGLLGMFETMQNP 60

WT2608_permease MNEKFARWNVLFIQYVKRDWKKIIVWVLGLGLFSGAYVPAFEEIAKGQGLLGMFETMQNP 60

WT1190_permease MNEKFARWNVLFIQYVKRDWKKIIVWVLGLGLFSGAFVPAFEEIAKGQGLLGMFETMQNP 60

WT1251_permease MNEKFARWNVLFIQYVKRDWKKIIVWVLGLGLFSGAFVPAFEEIAKGQGLLGMFETMQNP 60

WT1301_permease MNEKFARWNVLFIQYVKRDWKKIIVWVLGLGLFSGAFVPAFEEIAKGQGLLGMFETMQNP 60

WT1343_permease MNEKFARWNVLFIQYVKRDWKKIIVWVLGLGLFSGAFVPAFEEIAKGQGLLGMFETMQNP 60

************************************:***********************

WT1145_permease AMISMVGPTPIKIGTDYTLGAMYAQEMLLFCGLFAMIISALHVVSHTRKEEELGLTELVR 120

WT1402_permease AMISMVGPTPIKIGTDYTLGAMYAQEMLLFCGLFAMIISALHVVSHTRKEEELGLTELVR 120

WT2608_permease AMISMVGPTPIKIGTDYTLGAMYAQEMLLFCGLFAMIISALHVVSHTRKEEELGLTELVR 120

WT1190_permease AMISMVGPTPIKIGTDYTLGAMYAQEMLLFCGLFAMIISALHVVSHTRKEEELGLTELVR 120

WT1251_permease AMISMVGPTPIKIGTDYTLGAMYAQEMLLFCGLFAMIISALHVVSHTRKEEELGLTELVR 120

WT1301_permease AMISMVGPTPIKIGTDYTLGAMYAQEMLLFCGLFAMIISALHVVSHTRKEEELGLTELVR 120

WT1343_permease AMISMVGPTPIKIGTDYTLGAMYAQEMLLFCGLFAMIISALHVVSHTRKEEELGLTELVR 120

************************************************************

WT1145_permease SFRVGRQANSLAVISEMLLINLLLGLLIGGLMMSFGVKTIDAEGAFLFGGSIALAGIIGG 180

WT1402_permease SFRVGRQANSLAVISEMLLINLLLGLLIGGLMMSFGVKTIDAEGAFLFGGSIALAGIIGG 180

WT2608_permease SFRVGRQANSLAVISEMLLINLLLGLLIGGLMMSFGVKTIDAEGAFLFGGSIALAGIIGG 180

WT1190_permease SFRVGRQANSLAVISEMLLINLLLGLLIGGLMMSFGVKTIDAEGAFLFGGSIALAGIIGG 180

WT1251_permease SFRVGRQANSLAVISEMLLINLLLGLLIGGLMMSFGVKTIDAEGAFLFGGSIALAGIIGG 180

WT1301_permease SFRVGRQANSLAVISEMLLINLLLGLLIGGLMMSFGVKTIDAEGAFLFGGSIALAGIIGG 180

WT1343_permease SFRVGRQANSLAVISEMLLINLLLGLLIGGLMMSFGVKTIDAEGAFLFGGSIALAGIIGG 180

************************************************************

WT1145_permease VLALVMSQIMATSTGATGSTLSLIGLLYIVRAGTDVSNLDLSMFNPMGWIYLTYPFTKNN 240

WT1402_permease VLALVMSQIMATSTGATGSTLSLIGLLYIVRAGTDVSNLDLSMFNPMGWIYLTYPFTKNN 240

WT2608_permease VLALVMSQIMATSTGATGSTLSLIGLLYIVRAGTDVSNLDLSMFNPMGWIYLTYPFTKNN 240

WT1190_permease VLALVMSQIMATSTGATGSTLSLIGLLYIVRAGTDVSNLDLSMFNPMGWIYLTYPFTKNN 240

WT1251_permease VLALVMSQIMATSTGATGSTLSLIGLLYIVRAGTDVSNLDLSMFNPMGWIYLTYPFTKNN 240

WT1301_permease VLALVMSQIMATSTGATGSTLSLIGLLYIVRAGTDVSNLDLSMFNPMGWIYLTYPFTKNN 240

WT1343_permease VLALVMSQIMATSTGATGSTLSLIGLLYIVRAGTDVSNLDLSMFNPMGWIYLTYPFTKNN 240

************************************************************

WT1145_permease WLPLLFALIFSLVFTVLAFVLEEHRDMGAGYLPEREGRATAKKSLLSVPGLFFKINKGVM 300

WT1402_permease WLPLLFALIFSLVFTVLAFVLEEHRDMGAGYLPEREGRATAKKSLLSVPGLFFKINKGVM 300

WT2608_permease WLPLLFALIFSLVFTVLAFVLEEHRDMGAGYLPEREGRATAKKSLLSVPGLFFKINKGVM 300

WT1190_permease WLPLLFALIFSLVFTVLAFVLEEHRDMGAGYLPEREGRATAKKSLLSVPGLFFKINKGVM 300

WT1251_permease WLPLLFALIFSLVFTVLAFVLEEHRDMGAGYLPEREGRATAKKSLLSVPGLFFKINKGVM 300

WT1301_permease WLPLLFALIFSLVFTVLAFVLEEHRDMGAGYLPEREGRATAKKSLLSVPGLFFKINKGVM 300

WT1343_permease WLPLLFALIFSLVFTVLAFVLEEHRDMGAGYLPEREGRATAKKSLLSVPGLFFKINKGVM 300

************************************************************

WT1145_permease IGWLIAFVVMGAAYGSIYGDMQVFLGGNELMKQMFTQSGVSIEESFTATIMMVMIGLVTI 360

WT1402_permease IGWLIAFVVMGAAYGSIYGDMQVFLGGNELMKQMFTQSGVSIEESFTATIMMVMIGLVTI 360

WT2608_permease IGWLIAFVVMGAAYGSIYGDMQVFLGGNELMKQMFTQSGVSIEESFTATIMMVMIGLVTI 360

WT1190_permease IGWLIAFVVMGAAYGSIYGDMQVFLGGNELMKQMFTQSGVSIEESFTATIMMVMIGLVTI 360

WT1251_permease IGWLIAFVVMGAAYGSIYGDMQVFLGGNELMKQMFTQSGVSIEESFTATIMMVMIGLVTI 360

WT1301_permease IGWLIAFVVMGAAYGSIYGDMQVFLGGNELMKQMFTQSGVSIEESFTATIMMVMIGLVTI 360

WT1343_permease IGWLIAFVVMGAAYGSIYGDMQVFLGGNELMKQMFTQSGVSIEESFTATIMMVMIGLVTI 360

************************************************************

WT1145_permease LPIAVVNKLFAEETRLHLSQLYVTKITRGQLYWTTIFLAIFAGVVGIGLASAGLGGTAIS 420

WT1402_permease LPIAVVNKLFAEETRLHLSQLYVTKITRGQLYWTTIFLAIFAGVVGIGLASAGLGGTAIS 420

WT2608_permease LPIAVVNKLFAEETRLHLSQLYVTKITRGQLYWTTIFLAIFAGVVGIGLASAGLGGTAIS 420

WT1190_permease LPIAVVNKLFAEETRLHLSQLYVTKITRGQLYWTTIFLAIFAGVVGIGLASAGLGGTAIS 420

WT1251_permease LPIAVVNKLFAEETRLHLSQLYVTKITRGQLYWTTIFLAIFAGVVGIGLASAGLGGTAIS 420

WT1301_permease LPIAVVNKLFAEETRLHLSQLYVTKITRGQLYWTTIFLAIFAGVVGIGLASAGLGGTAIS 420

WT1343_permease LPIAVVNKLFAEETRLHLSQLYVTKITRGQLYWTTIFLAIFAGVVGIGLASAGLGGTAIS 420

************************************************************

WT1145_permease AMKNESTMDLTDFLAAGYNFLPSILFYIGLAALALGWLPKFGKVIYAYLGYSFALNYFGG 480

WT1402_permease AMKNESTMDLTDFLAAGYNFLPSILFYIGLAALALGWLPKFGKVIYAYLGYSFALNYFGG 480

WT2608_permease AMKNESTMDLTDFLAAGYNFLPSILFYIGLAALALGWLPKFGKVIYAYLGYSFALNYFGG 480

WT1190_permease AMKNESTMDLTDFLAAGYNFLPSILFYIGLAALALGWLPKFGKVIYAYLGYSFALNYFGG 480

WT1251_permease AMKNESTMDLTDFLAAGYNFLPSILFYIGLAALALGWLPKFGKVIYAYLGYSFALNYFGG 480

WT1301_permease AMKNESTMDLTDFLAAGYNFLPSILFYIGLAALALGWLPKFGKVIYAYLGYSFALNYFGG 480

WT1343_permease AMKNESTMDLTDFLAAGYNFLPSILFYIGLAALALGWLPKFGKVIYAYLGYSFALNYFGG 480

************************************************************

WT1145_permease ILDLPDWFSKTAIQSWIPRLPMEEFDGTIFAVITVISIVFLFVGYLGYKRRDMVEGA 537

WT1402_permease ILDLPDWFSKTAIQSWIPRLPMEEFDGTIFAVITVISIVFLFVGYLGYKRRDMVEGA 537

WT2608_permease ILDLPDWFSKTAIQSWIPRLPMEEFDGTIFAVITVISIVFLFVGYLGYKRRDMVEGA 537

WT1190_permease ILDLPDWFSKTAIQSWIPRLPMEEFDGTIFAVITVISIVFLFVGYLGYKRRDMVEGA 537

WT1251_permease ILDLPDWFSKTAIQSWIPRLPMEEFDGTIFAVITVISIVFLFVGYLGYKRRDMVEGA 537

WT1301_permease ILDLPDWFSKTAIQSWIPRLPMEEFDGTIFAVITVISIVFLFVGYLGYKRRDMVEGA 537

WT1343_permease ILDLPDWFSKTAIQSWIPRLPMEEFDGTIFAVITVISIVFLFVGYLGYKRRDMVEGA 537

*********************************************************

**Supplementary Figure 4. The promoter regions of narAB**

**A.**
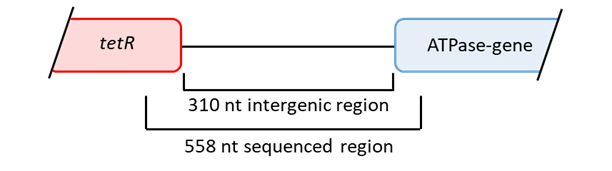


**B. Alignment Promoter region**

1402-upstr TTATAGAAAGTTTTTCCTACATTGGCCTTTTTGGCTACGGCATCGATCGTTAAATTCTCG 60

1190-upstr TTATAGAAGGTTTTTCCTACATTGGCCTTTTTGGCTACGGCATCGATCGTTAAATTCTCG 60

2608-upstr TTATAGAAGGGTTTTCCTACATTGGCCTTTTTGGCTACGGCATCGATCGTTAAATTCTCG 60

******** * *************************************************

1402-upstr ATTCCCGTATCTGTATTCATCAGATCAAAAGCAGCTCGTAAAACTTTTTTCTTTTTTTCT 120

1190-upstr ATTCCCGTATCTGTATTCATCAGATCAAAAGCAGCTCGTAAAACTTTTTTCTTTTTTTCT 120

2608-upstr ATTCCCGTATCTGTATTCATCAGATCAAAAGCAGCTCGTAAAACTTTTTTCTTTTTTTCT 120

************************************************************

1402-upstr TCTGTTCTTCGTTCAAAACCATCCATTAAAAATGCCTACTTTCTATAAAA-AGAACTTTT 179

1190-upstr TCTGTTCTTCGTTCAAAACCATCCATTAAAAATACCTACTTTCTATAAAAAAGAACTTTT 180

2608-upstr TCTGTTCTTCGTTCAAAACCATCCATTAAAAATACCTACTTTCTATAAAAAAGAACTTTT 180

********************************* **************** *********

1402-upstr TGTTTATTATAGTTCAAAACTAACTTCGTATATATTATATAGGAAAGAATACGTACTAAC 239

1190-upstr TGTTTATCATAGTTCAAAAATAGCTTCGTATATATTATATAGGAAATAATACGAACTAAC 240

2608-upstr TGTTTATCATAGTTCAAAAATAGCTTCGTATATATTATATAGGAAATAATACGAACTAAC 240

******* *********** ** *********************** ****** ******

1402-upstr AATGGAATTATAGGTCTAAATTTAAAATTATTTTTATTGACTGTTGAAATAAAAAAGAAT 299

1190-upstr AATGGAATCATAGGTTTAAATTTAAAATTATTTTTATTGACTGTTAAAATAAAAAAGAAT 300

2608-upstr AATGGAATCATAGGTTTAAATTTAAAATTATTTTTATTGACTGTTAAAATAAAAAAGAAT 300

******** ****** ***************************** **************

1402-upstr ATTATCGAATCAGTGAAAGAATTTTGGTTTGAAACAAAGTATAAAGGGGTATTTTATGAA 359

1190-upstr ATTATTGGAGCTGTAAAAGAACTTTGGTTTGAAACAAAGTATAAAGGGGTATTTTATGAA 360

2608-upstr ATTATTGGAGCTGTAAAAGAACTTTGGTTTGAAACAAAGTATAAAGGGGTATTTTATGAA 360

***** * * * ** ****** **************************************

1402-upstr TGATACATATTGCATTAGTTCATAAAAAAGGTGCGTTTTGTTCAGACTGGAAAGCTCATT 419

1190-upstr CGATACGTATTGTATTGGTTCATAAAAAAGGTGCGTTTTGTTCAGACTGGAAAGCTCGTT 420

2608-upstr CGATACGTATTGTATTGGTTCATAAAAAAGGTGCGTTTTGTTCAGACTGGAAAGCTCGTT 420

***** ***** *** **************************************** **

1402-upstr ATAAAATCATGTCAAAAGAAAAGGGAGTTATGTACCATGACAGAAATTGTAAAAGTACAA 479

1190-upstr ATAAAATCATGTCAAAAGAAAAGGGAGTTATGTACCATGACAGAAATTGTAAAAGTACAA 480

2608-upstr ATAAAATCATGTCAAAAGAAAAGGGAGTTATGTACCATGACAGAAATTGTAAAAGTACAA 480

************************************************************

1402-upstr GGCTTGCAAAAAAAATTTGGTAAATTCCAGGCGTTGAAAGATGTCTCATTCACAGTAAAC 539

1190-upstr GGCTTGCAAAAAAAATTTGGTAAATTCCAGGCGTTGAAAGATGTCTCATTCACAGTAAAC 540

2608-upstr GGCTTGCAAAAAAAATTTGGTAAATTCCAGGCGTTGAAAGATGTCTCATTCACAGTAAAC 540

************************************************************

1402-upstr GCCGGTGAAGTTGTTGGT 557

1190-upstr GCCGGTGAAGTTGTTGGT 558

2608-upstr GCCGGTGAAGTTGTTGGT 558

******************

| **Supplementary Table 1. Occurence of *narA* and *narB* in the isolates included in the study.** | | | | | | | |
| --- | --- | --- | --- | --- | --- | --- | --- |
| Sample | Strain | Result | | Sample | Strain | Result | |
|  |  | *narA* | *narB* |  |  | *narA* | *narB* |
|  |  | (476 bp) bp) | (303 bp) |  |  | (476 bp) | (303 bp) |
| 1 | WT15 | + | + | 10 | WT1251 | + | + |
| 2 | WT1137 | + | + | 11 | WT1301 | + | + |
| 3 | WT1145 | + | + | 12 | WT1343 | + | + |
| 4 | WT1147 | + | + | 13 | WT1366 | + | + |
| 5 | WT1155 | + | + | 14 | WT1402 | + | + |
| 6 | WT1165 | + | + | 15 | WT2608 | + | + |
| 7 | WT1174 | + | + | 16 | WT4826 | + | + |
| 8 | WT1179 | + | + | 17 | WT5252 | + | + |
| 9 | WT1190 | + | + | 18 | WT5432 | + | + |

**Supplementary Figure 5. Gel pictures showing PCR products of *narA***


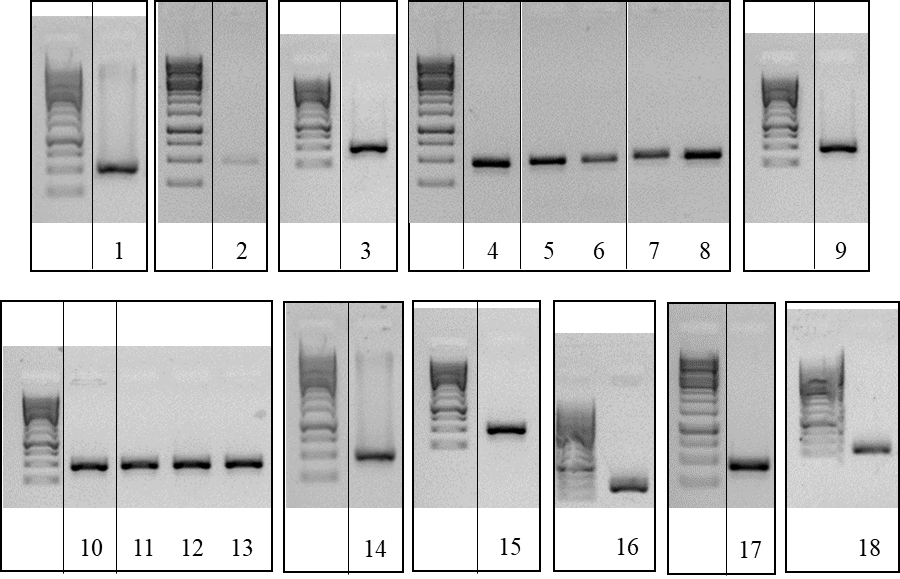


The gene *narA* was detected in the isolates described in Supplementary Table 1 by PCR using primers: The gel pictures within each frame correspond to the same gel, but the gel pictures have been assembled because the lanes were separated by samples not relevant for this study.

**Supplementary Figure 6. Gel pictures showing PCR products of *narB***


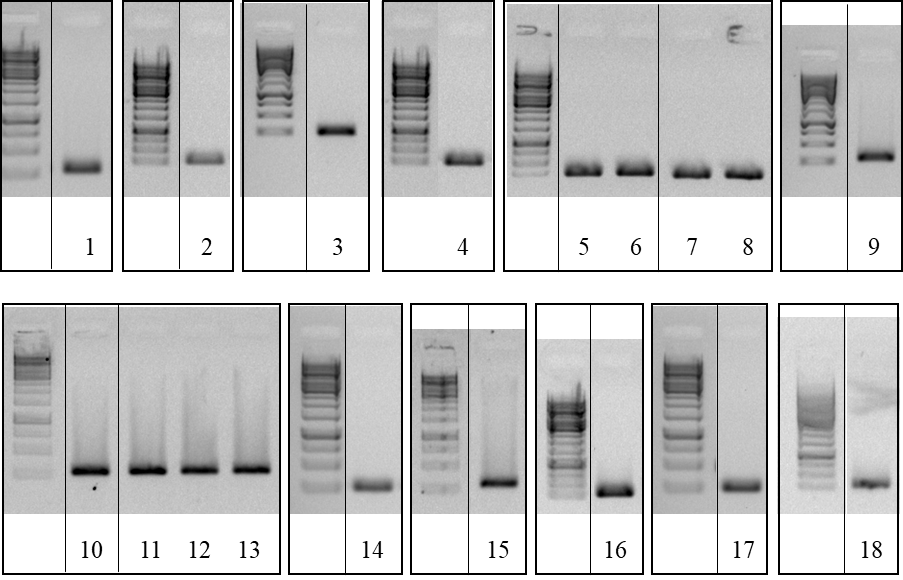


The gene *narA* was detected in the isolates described in Supplementary Table 1 by PCR using primers: The gel pictures within each frame correspond to the same gel, but the gel pictures have been assembled because the lanes were separated by samples not relevant for this study.

| **Supplementary Table 2. Detection of *narA, narB* and *vanA* in transconjugants, 64/3 and SE34** | | | | |
| --- | --- | --- | --- | --- |
| Number | Strain | *narA* (476 bp) | *narB* (303 bp) | *vanA* (121 bp) |
| 1 | Tc15 | + | + | - |
| 2 | Tc1137 | + | + | - |
| 3 | Tc1145 | + | + | - |
| 4 | Tc1147 | + | + | - |
| 5 | Tc1165 | + | + | - |
| 6 | Tc1174 | + | + | - |
| 7 | Tc1179 | + | + | - |
| 8 | Tc1190 | + | + | - |
| 9 | Tc1402 | + | + | + |
| 10 | Tc2608 | + | + | - |
| 11 | Tc4826 | + | + | - |
| 12 | Tc5252 | + | + | + |
| 13 | Tc5432 | + | + | - |
| 14 | 64/3* | - | - | - |
| 15 | SE34 | - | - | - |

Tc, transconjugant; numbers refer to the wild type isolate (WT) used as donor during conjugation; * 64/3 was used as recipient during conjugation; SE34 was the strain used in the characterization of the cloned nar-operon.

**Supplementary Figure 7: PCR products of *narA* in transconjugants, 64/3 and SE34**


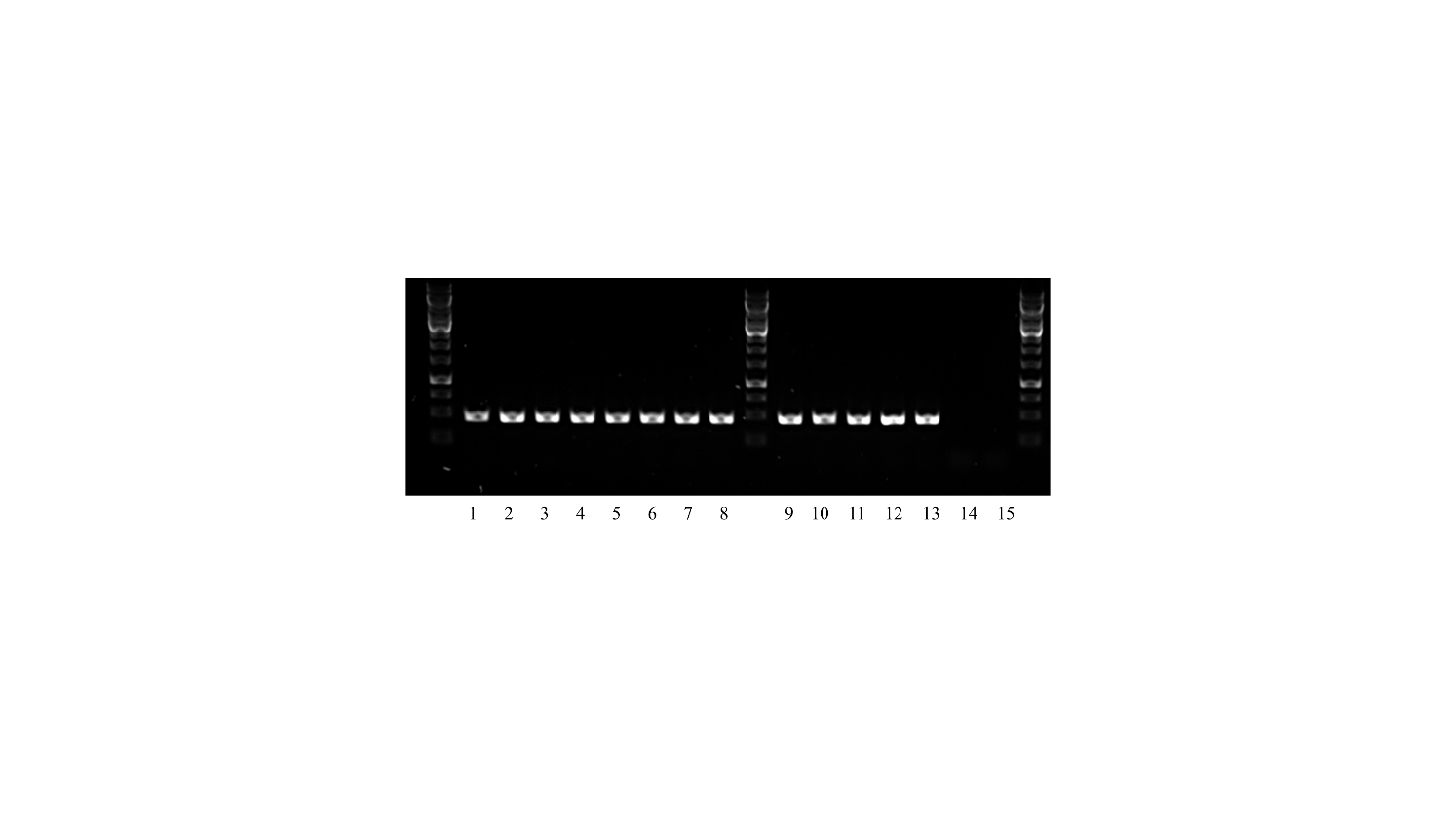


Numbers correspond to numbers in Supplementary Table 2.

**Supplementary Figure 8: PCR products of *narB* in transconjugants, 64/3 and SE34**


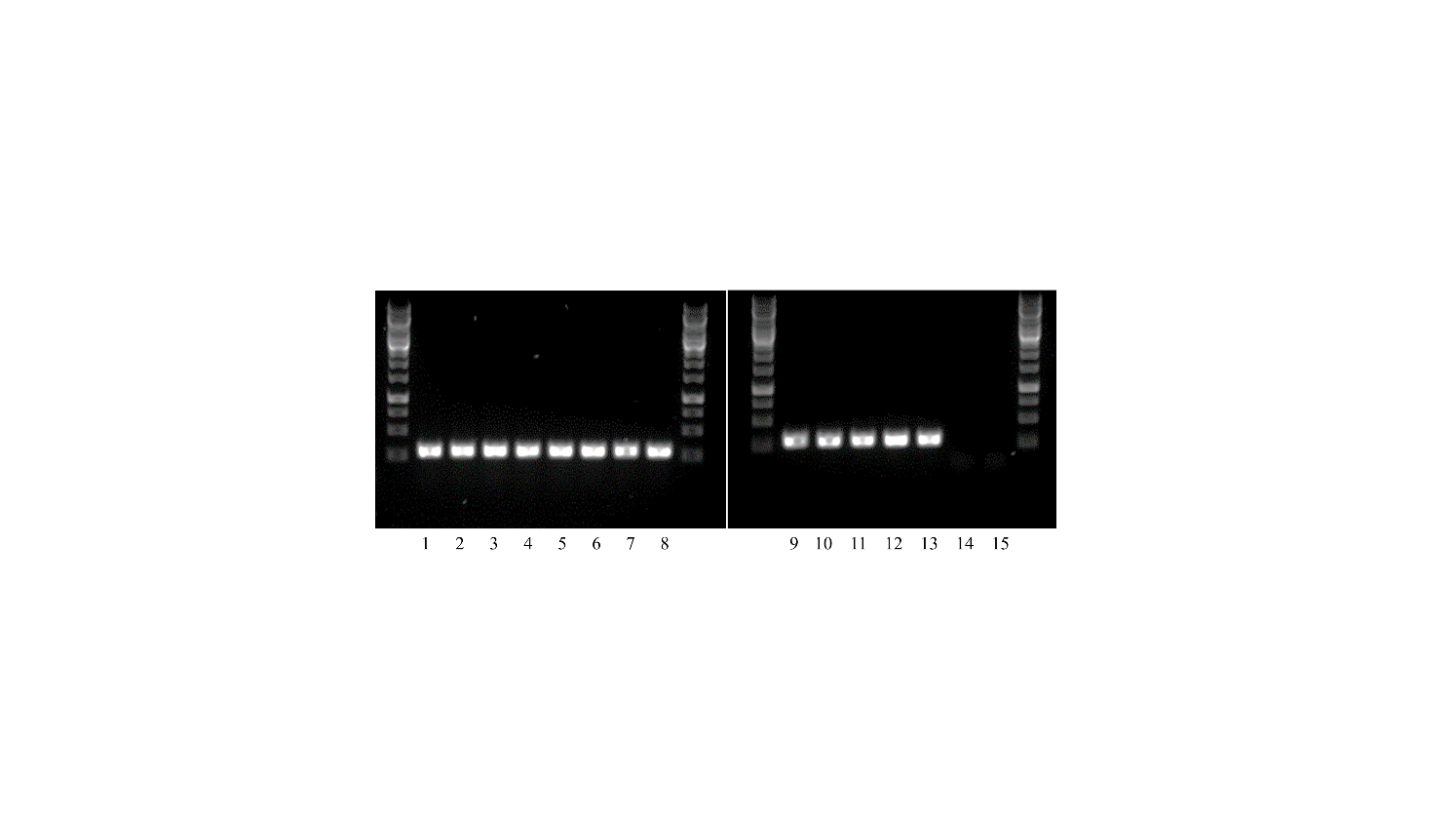


Numbers correspond to numbers in Supplementary Table 2.

**Supplementary Figure 9: PCR products of *vanA* in transconjugants, 64/3 and SE34**

*
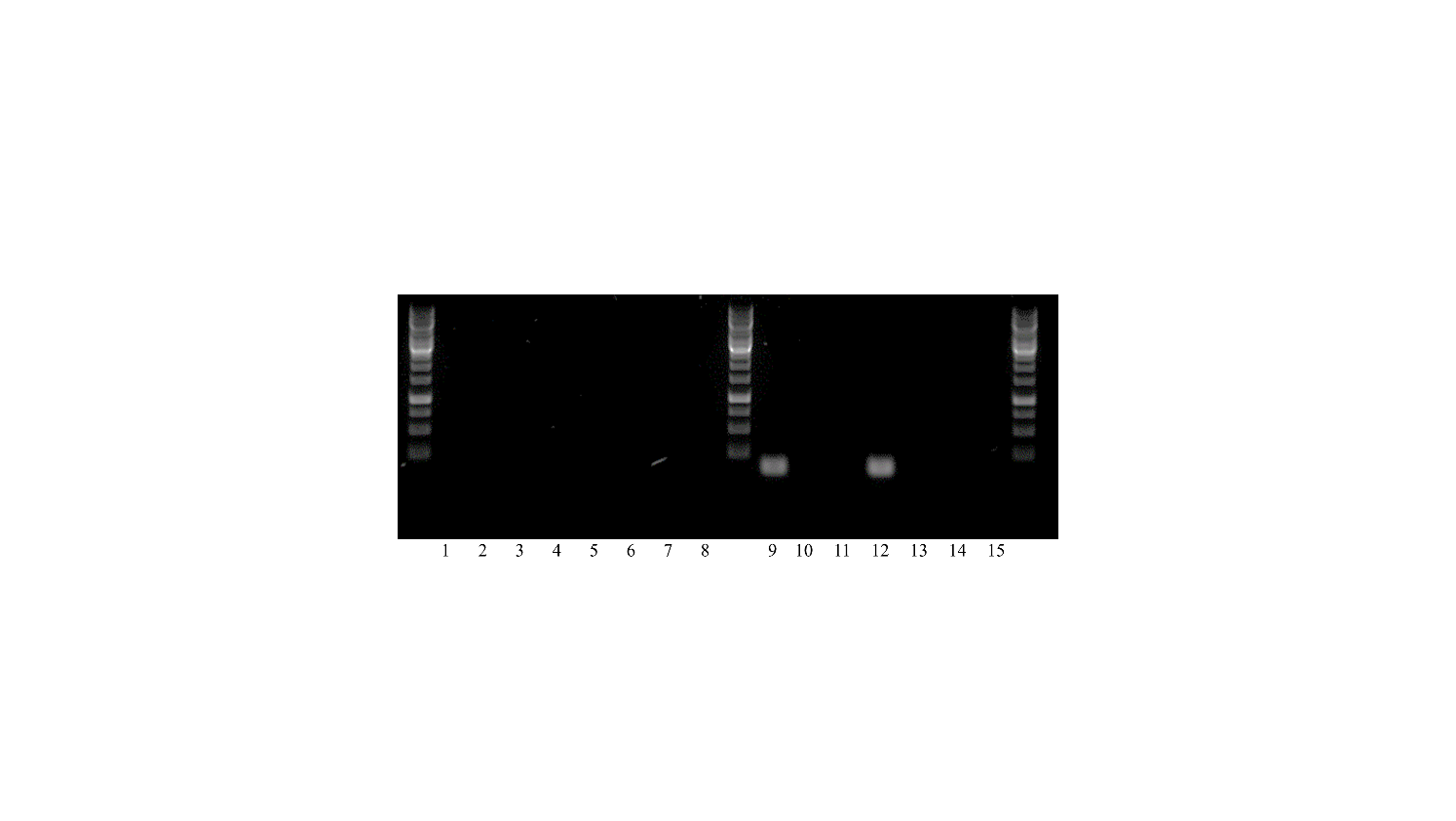
*

Numbers correspond to numbers in Supplementary Table 2.

| **Supplementary Table 3. Primers used in this study** | |
| --- | --- |
| **qPCR primers** | **Nucleotide sequence 5’ – 3’** |
| gyrA-RT-for | TGTACTACCTGCTCGCTTCC |
| gyrA-RT-rev | GCCGCTACTACCTCAGACAA |
| recA-RT-for | GTCGGGTTACAAGCACGTTT |
| recA-RT-rev | TCCCAAACATCACGCCTACT |
| Nar1-RT-for | TATCAAACTTCATGGCGGCG |
| Nar1-RT-rev | TGCAGCAATCAAACCGACTT |
| Nar2-RT-for | GCTGTTTTGCGGATTGTTCG |
| Nar2-RT-rev | TTGGCTTGTCGTCCTACTCG |
| **Cloning primers** |  |
| Nar-Reg-For | AAAT**GGATCC**CTATTGTCTCGGAGCGAT |
| Nar-Reg-Rev | AAAT**CTCGAG**TTAAGCGCCTTCTACCATAT |
| **Sequencing primers** |  |
| Nar-seq-rev1 | TGGCAGCAAAGAAAAGCTC |
| Nar-seq-rev2 | GACATCTTTCAACGCCTGG |
| Nar-seq-rev3 | ATCTGCTAATCGTTCAACTTC |
| Nar-seq-rev4 | ATCATTGCGAACAATCCGC |
| Nar-seq-rev5 | CTTGAAAAACAAACCAGGTAC |
| Nar-seq-rev6 | GCCTAGATAAGCATAGATTAC |
| Nar-seq-for | GAAACAAGACTGCATCTGAG |
| **Primers to detect *narA* and *narB*** |  |
| ABC_ATPase_For | TGTTCCTGGGGATGTTGCTC |
| ABC_ATPase_Rev | AGAGCGTCGCAAGTTTCTCA |
| ABCpermease_For | AGCTGCGTATGGCTCCATTT |
| ABCpermease_Rev | GCTGATGCTAAGCCAATGCC |
| **Primers to detect *vanA*** | |
| vanA Left | GGCTGCGATATTCAAAGCTC |
| vanA Right | CCACCGGCCTATCATCTTTA |
